# Trait-mediated speciation and human-driven extinctions in proboscideans revealed by unsupervised Bayesian neural networks

**DOI:** 10.1126/sciadv.adl2643

**Published:** 2024-07-24

**Authors:** Torsten Hauffe, Juan L. Cantalapiedra, Daniele Silvestro

**Affiliations:** ^1^ Department of Biology, University of Fribourg and Swiss Institute of Bioinformatics, 1700 Fribourg, Switzerland.; ^2^ Departamento de Paleobiología, Museo Nacional de Ciencias Naturales, Consejo Superior de Investigaciones Científicas, 28006 Madrid, Spain.; ^3^ GloCEE Global Change Ecology and Evolution Research Group, Departamento de Ciencias de la Vida, Universidad de Alcalá, 28801 Alcalá de Henares, Spain.; ^4^ Museum für Naturkunde, Leibniz Institute for Evolution and Biodiversity Science, Invalidenstraße 43, 10115 Berlin, Germany.; ^5^ Department of Biological and Environmental Sciences, Gothenburg Global Biodiversity Centre, University of Gothenburg, 40530 Gothenburg, Sweden.

## Abstract

Species life-history traits, paleoenvironment, and biotic interactions likely influence speciation and extinction rates, affecting species richness over time. Birth-death models inferring the impact of these factors typically assume monotonic relationships between single predictors and rates, limiting our ability to assess more complex effects and their relative importance and interaction. We introduce a Bayesian birth-death model using unsupervised neural networks to explore multifactorial and nonlinear effects on speciation and extinction rates using fossil data. It infers lineage- and time-specific rates and disentangles predictor effects and importance through explainable artificial intelligence techniques. Analysis of the proboscidean fossil record revealed speciation rates shaped by dietary flexibility and biogeographic events. The emergence of modern humans escalated extinction rates, causing recent diversity decline, while regional climate had a lesser impact. Our model paves the way for an improved understanding of the intricate dynamics shaping clade diversification.

## Teaser

Diversification analysis uncovers the reasons for the rise and fall of elephants and their extinct relatives.

## INTRODUCTION

Speciation and extinction constantly reshape the biosphere across evolutionary temporal scales. The shifting balance between these two processes dictates biodiversity patterns and their variation among clades and through time. These include turnover events retailoring regional ecosystem functioning (*1*, *2*), the emergence of global biogeographic patterns such as the latitudinal diversity gradient (*3*–*5*), the rise and fall of entire lineages in the sway with environmental disruption or increasing competition (*6*–*8*), and major biological transitions brought about by mass extinctions and subsequent diversity recoveries that virtually reset life on Earth (*9*). Crucially, speciation and extinction processes also play a leading role in fashioning biological disparity. When the pruning and sprouting of the tree of life are selective and favor certain properties of the species over others [i.e., species sorting or species selection; (*10*)], speciation and extinction become effective builders of evolutionary active trends that spur lineages into unexplored regions of the morphospace [(*11*–*13*) see “note added in proof”]. Thus, it does not come as a surprise that evolutionary research has long sought to link patterns of diversity to evolutionary processes (*14*–*16*). In doing so, a key goal of macroevolutionary research has been to quantify the effects of species properties on diversification (*17*–*19*), the effects of selective diversification on evolutionary trends (*20*), and the role of environmental forcing in this interplay (*21*, *22*).

Because these evolutionary processes leave their footprint on the fossil record and molecular phylogenetic trees (*23*), studying both sources of information has become a central research focus for evolutionary biologists. Quantitative assessments of diversification patterns through time and across clades have a long tradition in paleobiology (*24*, *25*) and have led to the development of a wide range of methods and models to infer speciation and extinction rates (*26*–*30*). Similarly, recognizing that molecular phylogenies to some extent encapsulate the signal of past diversification events (*31*–*33*) has fueled the development of diversification models tailored to analyze dated phylogenetic trees of extant taxa (*34*–*37*).

The realm of speciation and extinction inference has witnessed a blooming of numerous models that account for trait- and time-series dependence in the diversification process [e.g., (*8*, *18*, *28*, *38*–*44*)]. However, most diversification models only allow for the estimation of the effects of single predictors (e.g., traits or paleoenvironmental changes) on speciation and extinction rates. This is a critical limitation, as it is unlikely for just a single factor to have a constant leverage on speciation or extinction at macroevolutionary time scales. In addition, it has long been acknowledged that the causal relationship between phenotypic traits and diversification is expected to vary with changing environmental settings (*21*). For example, species with larger body size may be more likely to disappear during severe extinction events (*45*). However, as productivity and ecosystem functioning recover, niches related to larger body sizes could have greater diversification potential once smaller-sized guilds become saturated (*13*, *46*). While some of the models allow for a joint analysis of combined multiple factors (e.g., multiple traits or time variables), they typically assume that their effects are additive, independent, and constrained by monotonic (often linear) functions (*47*–*51*). Thus, any potential nonlinear effects and the shifting interplay between species attributes and paleoenvironmental changes in regulating speciation and extinction rates have proven elusive to quantify.

Here, we propose a flexible model for analyzing fossil occurrence data that allows time- and trait-dependent rate variation, thus integrating aspects of exploratory and hypothesis-driven models into a single cohesive framework. Our model is probabilistic and based on a stochastic birth-death process in which the rates are modulated through time and across lineages, using a flexible function of time, paleoenvironmental variables, and multiple categorical and/or continuous traits, along with their interaction. Our model does not assume a particular function linking traits and time to varying speciation and extinction rates a priori. Instead, the model estimates this function from the data through an unsupervised neural network. Leveraging methodologies from explainable artificial intelligence (xAI), we can estimate the effects of individual factors and rank their importance in shaping speciation and extinction. We applied our model to analyze the rich fossil record of proboscideans, examining the roles of paleoclimate, ecomorphology, biogeography, and spatiotemporal overlap with humans in shaping the diversification patterns of this mammalian order.

## RESULTS

### A new model of trait- and time-dependent diversification

We developed a Bayesian model to infer diversification dynamics from fossil occurrences based on a birth-death process coupled with a preservation and sampling process. Our approach builds upon the PyRate model (*30*) that uses a Bayesian algorithm to jointly sample the times of origination and extinction of all taxa in a dataset, as well as the speciation, extinction, and preservation rates. By implementing time-variable birth-death processes, PyRate can estimate variations in speciation and extinction rates through time. Our new model extends this approach by allowing speciation and extinction rates to vary both through time and at a lineage-specific level. This variation is inferred as a function of time itself, phylogenetic relatedness (for instance based on taxonomy or on a phylogenetic tree), and virtually any number of continuous and categorical phenotypic traits and environmental time-dependent variables. Instead of assuming a predefined response function linking these factors to speciation and extinction, we used an unsupervised neural network to model their correlation, thus allowing for nonlinear responses and interactions among the factors. We used a Markov chain Monte Carlo (MCMC) algorithm to sample the parameters of the neural network, from which lineage- and time-specific speciation and extinction rates are derived. These rates then feed into the likelihood function of a birth-death process that alongside the priors determines the acceptance probability in the MCMC algorithm. As in other PyRate implementations, the birth-death process is coupled with a model of preservation, allowing for variation of sampling rates through time and among lineages [see (*52*)].

Through the implementation of different algorithms adapted from xAI, we assessed the impact of the factors in shaping diversification dynamics. Specifically, we visualized the magnitude and shape of the inferred effect of traits and time-dependent variables on rates using partial dependence (PD) plots (*53*), which show the effect of a predictor while marginalizing over the others. We ranked the predictors by importance using three xAI approaches based on predictor permutations (*54*), marginal probabilities, and SHapley Additive exPlanation (SHAP) values (*55*). These metrics capture different aspects of the predictor’s impact: leverage on the birth-death likelihood (Eqs. 8 and 9), consistency of the direction of their effect, and effect size.

### Performance of the birth-death neural network model

We used simulations to benchmark the ability of our framework to accurately infer lineage-time–specific rates and correctly identify the factors causing rate variation. Specifically, we generated 100 fossil occurrence datasets for nine diversification scenarios. These scenarios accommodate speciation and extinction rates that are constant through time and equal among lineages (scenario 1), as well as rates that depend on time itself (e.g., through rate shifts), lineage-specific categorical or continuous traits, or a time series of paleotemperature and interactions among these factors (see fig. S1). Scenarios with time-dependent rates are interpreted as cases in which a time-varying factor drives the rate change. For instance, cases with rate shifts (scenarios 2, 3, and 8) could reflect changes in the speciation and extinction dynamics determined by a major environmental transition. Simulations portraying increasing extinction rates that ultimately reach speciation (scenario 5) capture the anticipated dynamics under a diversity-dependent competition model (*6*, *36*).

All datasets included multiple traits, time-dependent variables, and phylogenetic relatedness, even when they did not affect the rates. In simulations with rates changing at predefined times of shift (i.e., scenarios 2, 3, and 8), the times of rate shifts were considered as unknown and not provided as part of the datasets. Similarly, in scenario 9, the rates were dependent on a trait, which we, however, omitted from the dataset to evaluate the performance of the birth-death neural network (BDNN) model when missing the true predictors. In these cases, we expect the signal of rate variation to be captured either by time itself or by phylogenetic relatedness [here quantified as phylogenetic eigenvectors (*56*); see Materials and Methods] as proxies for the missing times of shift and traits.

**Fig. 1.**
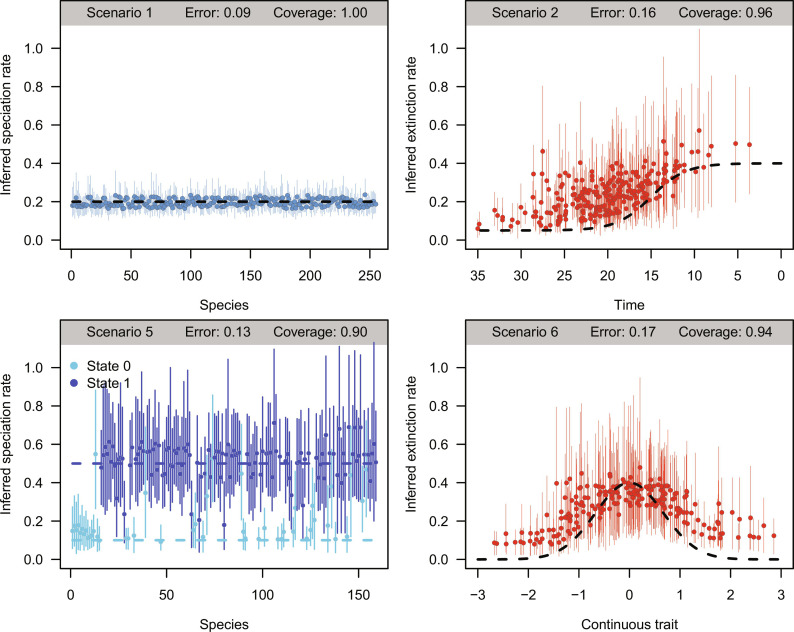
Comparing simulated and inferred lineage-time–specific rates. A single simulation under four diversification scenarios (settings listed in fig. S1) is displayed to exemplify the accuracy and coverage of rates inferred by the BDNN model. Dashed lines indicate simulated speciation and extinction rates. Dots display the means, and vertical solid lines display the 95% credible interval (CI) of the inferred rates. Accuracy was quantified by the median absolute percentage error between the simulated and inferred rates, and the coverage gives the share of lineages whose CI includes the simulated rate. The true simulated speciation rate was obtained from the ancestral lineage from which the lineage descended, from which the BDNN model infers the rate. All rates are given in units of events lineage^−1^ Myr^−1^.

The inferred lineage-time–specific speciation and extinction rates were accurate across all scenarios, with the median absolute relative errors being lowest in constant-rate simulations and ranging between 0.23 and 0.32 in most other scenarios (Fig. 1 and table S1). However, in simulations with piece-wise constant rates (scenario 3), the error increased to ∼0.38 to 0.66, as the BDNN model tended to smooth out the simulated instantaneous rate shifts (fig. S4D). Scenarios with rates dependent on categorical or continuous traits (simulations 5 and 6) yielded low errors around 0.23 to 0.25. More complex simulations involving the interacting effects of traits and time variables (scenarios 4, 7, and 8) still resulted in slightly lower accuracy with errors around 0.26 to 0.48, and similar accuracy was obtained even when the true predictors were omitted (scenario 9). With the Bayesian implementation of the BDNN model, we were able to calculate 95% credible intervals (CIs) or lineage-time–specific rates to assess how often they include the true values used to simulate their origination and extinction (i.e., coverage). The BDNN yielded a coverage ranging from 0.85 to 0.99 across most simulations, with lower values in scenario 3 (0.63 to 0.79; table S1). The coverage was around 0.85 in complex simulation scenarios with interactions between traits or with time dependence, while it exceeded 0.90 in simpler scenarios.

The accuracy of speciation and extinction rates inferred from the BDNN model substantially exceeded that of alternative models based on time-variable birth-death models (*52*) or the boundary-crosser method (*26*) in all simulations involving trait-dependent rates. For instance, in simulations featuring a categorical trait affecting speciation and extinction (scenario 5), the BDNN error was 0.25, compared to 0.57 based on alternative models (table S1). While the accuracy of BDNN rates was similar to that of alternative models in constant-rate simulations (scenario 1), the BDNN model was outperformed by time-variables models in simulations with sigmoidal time-varying rates or piecewise constant rates (scenarios 2 and 3). The coverage of the BDNN estimates was comparable or substantially higher than that based on alternative models across all simulations, except for scenario 3, where a birth-death model with rate shifts performed better.

### Assessment of significant rate variation

**Fig. 2.**
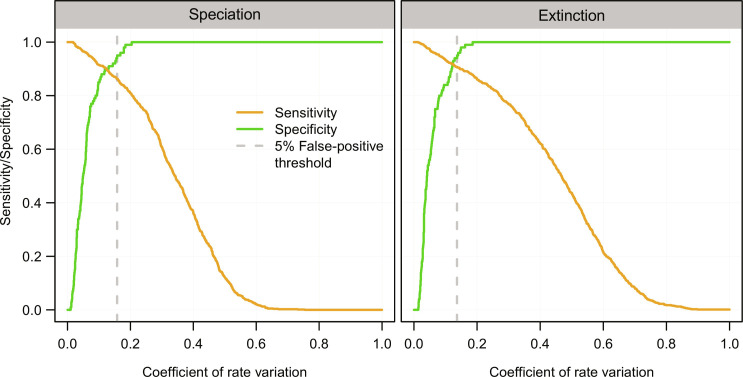
Power to find evidence of rate variation among species and over time. We quantified the coefficient of variation in inferred lineage-specific speciation and extinction rates, respectively. Across 100 thresholds, we calculated the proportion of correctly evidenced rate variation when simulated rates varied (i.e., mean sensitivity across scenarios 2 to 9) and erroneously inferred rate variation when, in fact, diversification under equal and constant rates was simulated (i.e., specificity for scenario 1). Dashed vertical lines display the thresholds for speciation and extinction above which a constant-rate model was rejected with 95% specificity.

We used the coefficient of variation in the estimated lineage-time–specific rates (see Materials and Methods; Fig. 2) to determine whether it exceeded what would be expected under a constant birth-death process. To this aim, we used the simulations generated under a constant birth-death process (scenario 1) to establish a baseline coefficient of variation, above which we rejected a constant birth-death model in favor of a BDNN model. We chose a baseline corresponding to a 95% specificity, i.e., leading to the erroneous rejection of a constant birth-death model in 5% of the simulations. On the basis of these thresholds, the sensitivity (percentage of correct rejection of the constant-rate model in favor of a BDNN model) averaged 86.3% for speciation and 90.8% for extinction. The sensitivity varied among scenarios, exceeding 80% in most simulations (fig. S2). It was lowest in scenario 9, where the trait driving rate variation was omitted from the analysis. The inferred rate variation overlapped with the simulated one, with a slight tendency to underestimate the true coefficient of variation in lineage-time–specific rates (fig. S3).

### Identification of the predictors of rate variation

**Fig. 3.**
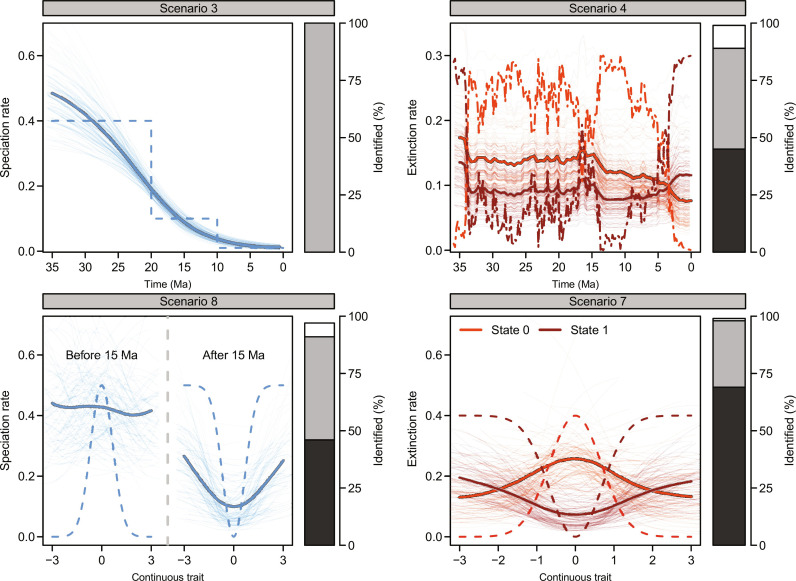
Inferred effects of selected predictors on speciation and extinction rates. PD plots showing the inferred effect of traits and time-dependent variables that actually influenced simulated speciation and extinction rates while marginalizing over predictors without a simulated impact. Dashed lines visualize the simulated effect, transparent lines show the 100 simulated replicates, and the thick solid line represents the average across them after locally estimated scatterplot smoothing (loess). Height of the barplots displays the proportion of simulations that exceeded our threshold to detect rate variation (i.e., rejecting the constant-rate model). The share of dark gray indicates how often the correct traits or time-dependent variables were correctly identified (when included among the predictors), and the white proportion shows incorrectly identified predictors. Light gray displays cases where the predictive time series was not included and instead time or phylogenetic relatedness was detected (scenarios 3 and 8) or only one of the two predictors was found (scenarios 4 and 7). Unit of rates are events lineage^−1^ Myr^−1^.

We performed several postprocessing steps to estimate the ability of the BDNN model to identify the factors determining rate variation. First, we visualized the magnitude and shape of the effects of traits and time-dependent variables on rates using PD plots. These showed a good match between the simulated and inferred relationship of rates with traits and time-dependent variables (Fig. 3 and fig. S4), even in the case of nonmonotonic effects, such as bell-shaped links between continuous traits and rates, and interactions among traits and time-dependent variables. While the shape of the effects was correctly inferred, albeit with variation among simulations, their magnitude was generally underestimated. For instance, the 5-fold difference in rate between the two states of a categorical trait (scenario 5; fig. S4E) was accurately inferred for extinction [mean across 100 replicates: 4.1; 95% confidence interval: 2.2 to 6.5], whereas we only found a 1.9-fold difference (1.3 to 2.8) in the case of speciation. The magnitude of rate variation attributed to individual predictors was therefore lower than the heterogeneity detected among marginal lineage-time–specific rates (Fig. 1). This is because PD plots isolate the effect of single predictors, thus removing some of the variation attributed by the model to different factors.

We then ranked the relative importance of traits and time-dependent variables according to three metrics based on permutations, marginal probabilities, and SHAP values. Using a consensus ranking method (*57*) among these metrics proved to be more robust than any individual metric alone (fig. S5). In scenarios where rates varied through time on the basis of times of rate shift not explicitly included in the analyses (i.e., scenarios 2 and 3), time itself was identified as the most important predictor in 76 to 100% of the simulations where a constant-rate model was rejected (fig. S5). In up to 20% of the simulations, the most important factor was identified as one of the phylogenetic eigenvectors, which inherently reflect both phylogenetic relatedness and time. When rate variation was driven by a trait omitted from the analysis (scenario 9), the signal was mostly attributed to time or phylogenetic eigenvectors (75 to 85% of the simulations), although the constant-rate model was rejected in this case only in about 50% of the cases (fig. S2). In scenario 4, where rates depend on both paleotemperature and a discrete trait, the former ranked among the top two predictors in 96 to 97% of the cases, while the discrete trait was correctly identified only in 26 to 46% of the simulations. In 40 to 57% of the simulations, the trait signal was attributed to phylogenetic eigenvectors, highlighting the difficulty to distinguish between the effect of a trait evolving along the phylogeny and the phylogeny itself. In cases where one or more traits drive the changes in speciation and extinction rates (scenarios 6 and 7), the correct trait(s) ranked as the top predictors in 21 to 90% of the simulations, with time or phylogenetic eigenvectors being favored in most other cases. In other scenarios where a trait affects the rates with variable effects through time (scenarios 5 and 8), the correct trait ranked among the top two predictors in 49 to 100% of the simulations, while time or phylogenetic eigenvectors are found among the top two in nearly all cases.

Overall, we found that time and phylogenetic eigenvectors can effectively capture the signal of rate variation, even when the true predictors are included, but most frequently when they are not. This indicates that they provide valuable null hypotheses that allow for rate variation without implying a specific factor. As a result, the probability of erroneously identifying a trait as the top-ranking predictor when it did not determine rate variation (i.e., a false positive), was 3.1%.

### The drivers of proboscidean diversification

We used the BDNN model to analyze the dynamics and drivers of speciation and extinction in proboscideans based on their rich fossil record. We used a published dataset consisting of 2118 fossil occurrences and detailed ecomorphological information for 175 proboscidean species (*58*). It includes 17 traits (e.g., body size, tusk morphology, and dental features), which were summarized through nonmetric multidimensional scaling (NMDS) into a two-dimensional ecomorphospace that captured 93% of the total ecomorphological variation. In addition, we incorporated information on geographic distribution (i.e., presence in Africa, Eurasia, Americas, or islands), regional paleotemperature, and paleovegetation (approximated as a binary variable reflecting the time of emergence of open-habitat grasslands in each geographic area). Furthermore, we used the flexibility of the BDNN model to incorporate phylogenetic information (summarized into two eigenvectors) to account for the nonindependence of traits and to capture the possible effects of unobserved predictors. Last, we included the spatiotemporal overlap with early humans [coded for African and Eurasian species between the Early and Late Pleistocene, 1.8 million years (Ma) to 129 thousand years (ka)] and with modern humans (after 129 ka in Africa and Eurasia, and in the Holocene 11.7 ka in the Americas) as a potential predictor for extinction. As these time boundaries were determined on the basis of geological stages (following the binning used for the other paleoenvironmental variables), they can only approximate the timing of human evolution and expansion out of Africa.

**Fig. 4.**
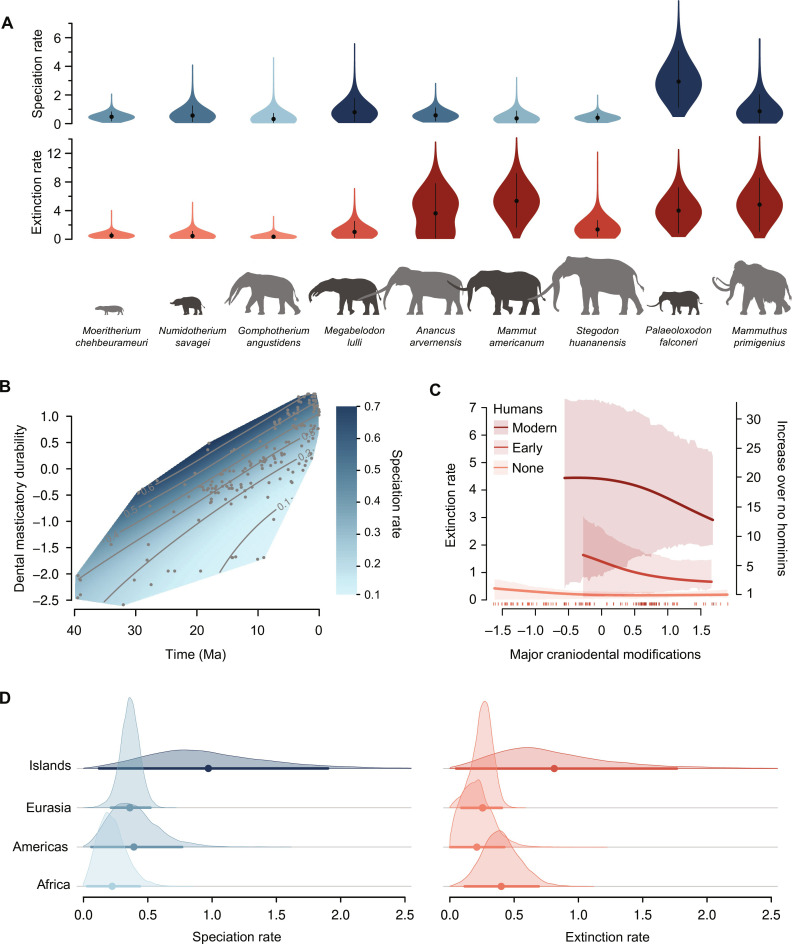
Analysis of the proboscidean fossil record using the BDNN model. (**A**) The inferred species-specific speciation and extinction rates show substantial heterogeneity, as showcased by the nine species displayed here. (**B** and **D**) Changes in speciation rates are mostly driven by time, geography, and an ecomorphological trait related to diet, with the highest rates found in the Oligocene and Miocene species with generalist diet, and with a consistent increase in island versus continental species. (**C**) Extinction rates were primarily modulated by the spatiotemporal overlap with early and modern humans, with additional effects of an ecomorphological trait related to mandible and tusk shapes, and (D) with higher extinction in island species. Unit of rates are events lineage^−1^ Myr^−1^. Silhouettes are made available on PhyloPic.

The coefficients of variation among the estimated species-time–specific rates were 0.80 for speciation and 1.14 for extinction. These strongly exceeded the thresholds of 0.20 and 0.25, respectively, which were inferred from simulations based on constant-rate models (see Materials and Methods), thus indicating compelling evidence for significant rate variation. We summarized the species-time–specific rates to obtain overall rate trajectories through time and found that speciation rates declined fourfold between the Late Eocene (40 Ma) and the end of the Miocene (5.3 Ma), followed by a rate increase of similar magnitude between the Pliocene and the Pleistocene (fig. S6A). Extinction rates were overall stable until the Early Pleistocene (1.8 Ma), when they increased fourfold by the end of the Pleistocene, followed by a further sixfold increase in the Holocene (11,700 years ago; fig. S6B). We also found evidence of strong heterogeneity in speciation and extinction rates among species. For instance, in the Pleistocene, we inferred a speciation rate of 2.91 (CI: 1.06 to 4.92) for the Sicilian dwarf elephant *Palaeoloxodon falconeri* (Fig. 4A), which is 8.3 times higher than the rate of the Eurasian *Stegodon huananensis* (mean: 0.35; CI: 0.10 to 0.65). Similarly, the estimated extinction rates varied 20-fold between the recently extinct woolly mammoth (*Mammuthus primigenius*; 3.91, CI: 0.42 to 7.35) and the Neogene species *Gomphotherium angustidens* (0.20, CI: 0.02 to 0.46).

We found that the three most important factors affecting speciation rate were an ecomorphological trait, time, and geographic distribution (Fig. 4, table S2, and data S1). The partial dependent plot shows that the emergence of phenotypes with enhanced dental masticatory durability (NMDS1) led to an increase in speciation rate (Fig. 4B). The BDNN model further evidenced that the correlation between NMDS1 and speciation rates became stronger over time. While the breadth of this ecomorphological trait is similar in the Early Oligocene and in the Late Miocene (∼30 and ∼7 Ma, respectively), the change in speciation rate attributed to this trait increased from 7- to 16-fold during this time frame. Yet, our proxy for the inception and expansion of open-habitat grasslands ∼20 to 16 Ma was not identified as a vital factor in proboscidean diversification. Last, species occurring on islands showed, on average, a ∼2.5 times higher speciation rate than those in the Americas and Eurasia and ∼4.2-fold increase over African species.

We estimated the proboscideans extinction rates to be most strongly affected by the overlap with humans, followed by more limited effects of geographic distribution and ecomorphology linked with tusk and mandible shapes (Fig. 4C and table S2). On the basis of partial dependent plots, the spatiotemporal overlap with early humans starting around 1.8 Ma was associated with a 5-fold increase in extinction rate, while the effect of modern *Homo sapiens* in the Late Pleistocene and Holocene was linked with a 17-fold increase. Geography was connected with a 2.7-fold variation, with island species subjected to higher extinction rates. Major craniodental morphologies (NMDS2) were identified as the third predictor and associated with a 3.3-fold variation in extinction rate, with the highest rate associated with long mandibles, cusped browsing-adapted molars, and shovel-like lower tusks (Fig. 4C). Notably, regional paleotemperature ranked fourth among the predictors (fig. S7), with highest extinction rates linked with low temperatures, while time itself was not found to be an important predictor.

To evaluate the robustness of our empirical results regarding the selection of predictors, we repeated the analyses on the basis of a subset of them. Specifically, we omitted open-habitat grasslands, replaced the ecomorphological traits with discretized body mass, and simplified biogeography to a binary insularity trait. Despite these modifications, we obtained similar predictions, with consistent species-time–specific rates inferred across 98% of the species (*R*^2^ = 0.81 for speciation and *R*^2^ = 0.86 for extinction; fig. S8). Insularity and humans were still remained among the most important drivers of rate variation (table S3 and data S2). However, we found that body mass alone did not capture the signal encoded in the NMDS axes and was not identified as an important predictor, emphasizing that cranial and masticatory adaptations had a greater impact on proboscidean diversification than size alone. Instead, this analysis identified both phylogenetic eigenvectors among the top predictors of rate changes, reflecting the relatively strong phylogenetic signal in the distribution of ecomorphologies within the clade (*58*).

## DISCUSSION

### A Bayesian unsupervised neural network model of diversification

Our understanding of macroevolutionary dynamics relies on our ability to accurately estimate speciation and extinction rates and the environmental variables and life-history traits that may drive them. Although it is likely that multiple factors jointly contributed to shaping clade diversity through time, most macroevolutionary analyses of diversification have focused on individual predictors, e.g., with diversity-dependent speciation (*6*), time-dependent rates (*35*), or paleotemperature effects on diversification (*41*). Although some models can incorporate multiple predictors of speciation and extinction, they typically make simplistic assumptions due to methodological constraints. For instance, they often assume monotonic and additive effects without interactions and allow for rate variation either through time (*48*) or among lineages (*49*, *59*). We present here the BDNN model, which relaxes many of the assumptions underlying alternative diversification models, simultaneously allowing for rate variation among lineages and through time. The BDNN approach allows us to jointly infer the effects of multiple predictors on speciation and extinction from fossil occurrence data while accounting for preservation biases. Our simulations showed that the model performs well under a broad range of evolutionary scenarios, including nonmonotonic effects of time variables and traits, and their interaction. Across a wide range of simulations, we noted that the BDNN model consistently outperformed alternative approaches based on birth-death processes with rate shifts (*53*) and the boundary-crosser method (*27*), both in terms of higher accuracy and better coverage. When the generating process was a birth-death with instantaneous rate shifts (scenario 3), then the birth-death model with shifts, expectedly, and the boundary-crosser method performed better than the BDNN. Conversely, in scenarios where the rates were modulated by a continuous trait omitted from the analyses (scenario 9), we found that the BDNN model achieved lower sensitivities, i.e., conservatively favoring a constant birth-death model in a larger fraction of simulations. Even in this case, however, it reached better accuracy compared to the alternative birth-death and boundary-crosser models. These results, along with previous simulation studies exploring the performance of different methods to infer speciation and extinction rates from fossil data (*52*, *60*–*62*), suggest that the BDNN model can lead to substantial improvement of the estimated rates across a range of evolutionary scenarios.

The BDNN model combines hypothesis-driven predictors, such as trait-mediated speciation rates or climate-related extinction, with rate variation that is agnostic about biological expectations, such as time-varying rates and variation based on phylogenetic relatedness. This integration is important because the unrealistic assumption of a constant-rate process as the null expectation, typical of many birth-death models (*8*, *18*, *48*), can lead to the spurious identification of traits or environmental predictors of diversification dynamics (*42*, *63*, *64*). Although the complex parameterization of the BDNN model makes it unfeasible to explicitly model test and assess the significance of each predictor, for instance, through Bayes factors, we showed that explainable AI tools can be repurposed to identify the most important factors affecting diversification. This reinforces recent findings evidencing that SHAP values derived from regression tree models can identify traits linked to survival across mass extinction events (*65*). While these methods do not currently allow us to rule out any predictors, we found that they can accurately quantify their relative importance and with comparable performance for speciation and extinction. Similarly, high levels of accuracy were recovered across different simulation settings, including scenarios with interacting traits and time-dependent variables. As with multiple regression models, we can expect the BDNN to have limited power in disentangling the effects of highly correlated traits or time series. In some of our simulations, phylogenetic eigenvectors, which are inherently correlated with predictive evolutionary traits, ranked among the top factors. Therefore, it is important to carefully select biologically meaningful variables and traits when setting up the analysis (*64*) and to account for time and phylogenetic relatedness, either through phylogenetic eigenvectors or taxonomy, as a way to accommodate unobserved predictors (*42*, *66*).

Neural networks have been recently used to predict speciation and extinction rates from fossil or phylogenetic data under simple age- or trait-dependent models (*67*–*69*). These methods were applied within a supervised learning framework, in which (i) labeled training datasets (fossil occurrences or phylogenies) are simulated under known speciation and extinction rates; (ii) neural networks are trained to predict the generating rates from features describing the simulated data; and (iii) the trained models are then applied to the unlabeled empirical dataset, where the rates are unknown. While these approaches have yielded accurate results, their scalability to more complex scenarios involving rate variation and dependence on multiple traits and time variables, as tested under our BDNN model, would likely require generating training data under an unfeasibly wide range of simulated scenarios. In contrast, the model presented here is unsupervised, i.e., it does not rely on a labeled training dataset. Instead, it infers the rates of speciation and extinction based on the likelihood of the empirical (unlabeled) fossil data under a birth-death and preservation model. This framework allows us to infer diversification dynamics and how they are shaped by different predictors without the need to a priori define the range of possible rate variation and correlations.

While the use of a neural network offers high flexibility in terms of which and how many predictors can be analyzed and how they affect the rates, it is likely to represent an overparameterized model, which includes several parameters (weights) that are not contributing significantly to explaining the data. This is particularly important in a frequentist use of neural networks generally applied to supervised learning tasks, where the optimization of the weights must be counterbalanced by regularization techniques, e.g., using dropout, and by rules to prevent overfitting, e.g., stopping the optimization on the basis of the performance of the model on a validation set (*70*). However, in Bayesian neural networks, the priors on the weights have a regularizing effect, thus effectively mitigating the risk of overfitting in the context of supervised learning (*71*). This was further facilitated in our implementation by the introduction of a regularizing output layer (see Materials and Methods, Eqs. 6 and 7). As a result of the regularization, our model was able to provide highly accurate and precise rates even when the generating process was constant (table S1 and Fig. 1). Another advantage of Bayesian neural networks over their frequentist counterpart is their ability to provide direct estimates of the uncertainty around the parameters. By sampling weights from their joint posterior distribution rather than relying on point estimates, we are able to obtain posterior estimates of speciation and extinction rates along with their 95% CIs. Thus, despite the complexity of the BDNN parameterization, overparameterization of the Bayesian neural network does not lead to spurious results and is mostly reflected in inflated CIs around rates, as opposed to significant deviations from the true values (Fig. 3 and fig. S4). Given that Bayesian CIs in birth-death models tend to decrease with larger datasets (*30*), the use of a complex model such as the BDNN, especially in conjunction with multiple predictors, is therefore most suitable for clades with a rich fossil record.

### The multifaceted macroevolution of proboscideans

Our analysis of the proboscidean fossil record revealed that the diversification of this clade was shaped by multiple factors and their interactions. Although the identified factors are broadly in agreement with previous findings based on independent analyses of some of them separately (*58*), our BDNN model allowed us to rank them and assess their interactions. Specifically, speciation within the clade was predominantly driven by dietary traits [as previously estimated; (*58*)], but our model also revealed a previously unidentified time effect that amplified this trait-driven variation through time. Dietary adaptation is likely a relevant driver of speciation across animal clades, unlocking access to new resources and enabling differentiation (*47*, *72*–*74*). Although we found no evidence of diversification shaped by major vegetation changes (encoded here as the emergence of the first open-habitat grasslands), it is likely that our proxy lacked sufficient spatial and temporal levels of detail to fully capture the extent of biome changes on proboscidean diversification. However, our model revealed that the link between traits associated with higher dietary flexibility and speciation rates strengthened toward the end of the Neogene, as lineages with highly durable dentition (characterized by high-crowned molars with a high number of enamel ridges) and derived masticatory modes evolved. We interpret this trend as an indirect effect of habitat change on this clade and hypothesize that environmental forcing could have played a key role in building evolutionary trends. This could have happened initially by fueling phenotypic innovation through efficient population-level selection (*75*) and then by exacerbating the proliferation of lineages featuring the most recent, derived morphologies, as also inferred for other ungulate groups (*74*).

The biogeographic aspect, particularly the association with islands, was found to be important for both speciation and extinction, in line with findings in various other taxa such as mammals, birds, squamates (*76*–*79*), and many other clades [e.g., *80*)]. It is predicted that these effects may arise because of dispersal followed by isolation, resulting in speciation, while smaller populations and limited resources on islands lead to higher extinction rates (*81*). The increased vulnerability of species on islands has greatly amplified human effects on island biodiversity (*79*).

The primary driver of proboscidean extinction was inferred to be the overlap with the human lineage, aligning with the growing body of evidence indicating humans’ severe impact on recent extinctions and on megafauna in particular (*79*, *82*–*86*). Our model, and the use of PD plots, allows us to single out the effect of humans after accounting for all other factors, suggesting that the estimated 5- to 17-fold rate increase attributed to early and modern humans is not influenced by other factors considered here (e.g., climate change, geographic distribution, or ecomorphological traits). We found that while humans exhibit the greatest impact in the past ca. 120,000 years, our results also point to a weaker yet significant influence of the human lineage at earlier times, thus supporting other studies suggesting a long-lasting detrimental anthropogenic effect on biodiversity (*79*, *87*–*89*).

In our analyses, climate change ranked fourth among our predictors, suggesting a potential impact of cooling leading to a higher extinction, albeit with a comparatively small inferred effect. There are, however, many additional aspects, such as seasonality, precipitation, and small-scale climate variation, that we cannot incorporate in our analyses because of limitations in the available data, especially in the deeper past. Similarly, assessing the exact timing and spatial distribution of vegetation changes [such as C4 grass expansion (*90*)], and the extent of competition from the diversification of other herbivore lineages such as bovids remains difficult (*58*). Thus, the effect of climate, environmental changes, and biotic interactions on proboscidean diversity are likely to be more nuanced than we can detect here with the available data. With the continuous growth in as the availability of digitized fossil occurrences and morphological traits (*91*–*93*), alongside the progress in modeling and measuring paleoclimate and the evolution of paleoenvironments (*94*–*96*), we expect to gain an increasingly accurate picture of past and recent evolutionary dynamics. In this context, our BDNN model paves the way for a powerful assessment of the processes of speciation and extinction, as well as the forces driving the rise and fall of clades.

## MATERIALS AND METHODS

### The PyRate framework

Our model is based on the Bayesian framework implemented in PyRate to analyze fossil occurrence data (*52*). The framework models the occurrence data as the result of a nonhomogeneous Poisson process representing preservation and sampling and a birth-death process describing the distribution of origination and extinction times. Using an MCMC algorithm, PyRate estimates from the joint posterior distribution: (i) the preservation rates and their variation through time and across lineages, (ii) the times of origination and extinction of all lineages, and (iii) the speciation and extinction rates.

Different birth-death processes can be used to test for rate variation through time based on piecewise constant models (*30*) or correlation with time-dependent variables (*84*). Alternatively, rate variation among lineages can be estimated through correlations with a lineage-specific trait or dependent on the states of a categorical trait (*49*). However, these models currently cannot be mixed to, for instance, include time- and trait-dependent effects, and assume simple linear or exponential correlations between the rates and the predictors (time series or traits) with no interactions.

### The BDNN model

We developed a model that can accommodate multiple time- and trait-dependent effects and their interactions, without limiting the effects to simple functions. Traits can be of different nature, such as quantitative, ordinal, and categorical traits. Categorical variables with more than two states are expected to be one-hot encoded into binary traits. The rate at time *t* for lineage *i* is modeled through a feed-forward neural network taking as input time and the characters and returning a lineage-time–specific speciation rate λ^(*i*, *t*)^ as output. An independent network with the same architecture is used to obtain lineage-time–specific extinction rates μ^(*i*, *t*)^. The input of the neural network is a matrix of size *J*, where *J* is the number of predictors $x^{\left(i,t\right)}=\left\{t,c_{1}^{\left(i\right)},c_{2}^{\left(i\right)},\ldots \right\}$ assigned to a species *i* at time *t*, which include time itself as well and categorical and continuous traits and time-dependent variables [*c*^(*i*)^]. The first hidden layer of the network *h*^(1)^ is a matrix of size *L*^(1)^

$$h_{l}^{\left(1\right)}=g\left[\sum_{j=1}^{J}‍x_{j}^{\left(i,t\right)}W_{jl}^{\left(1\right)}\right]$$ (1)

where *j* ∈ {1, …, *J*} identifies the predictor, *l* ∈ {1, …, *L*^(1)^} indicates the hidden nodes, and *W*^(1)^ is a matrix of *J* × *L*^(1)^ weights. We indicate with *g*( · ) the tanh activation function, which has been shown to perform well in small neural networks (*97*), here approximated to

$$g\left(z\right)=1-\frac{2}{e^{2x}+1}$$ (2)

Similarly, the second hidden layer is a matrix of size *L*^(2)^

$$h_{k}^{\left(2\right)}=g\left[\sum_{l=1}^{L^{\left(1\right)}}‍h_{l}^{\left(1\right)}W_{lk}^{\left(2\right)}\right]$$ (3)

where *k* ∈ {1, …, *L*^(2)^} indicates the hidden nodes of the second layer and *W*^(2)^ is a matrix of *L*^(1)^ × *L*^(2)^ weights. A third layer takes *h*^(2)^ as input and returns a species-time–specific baseline rate, e.g., for speciation rate

$$\lambda_{b}^{\left(i,t\right)}=\sigma \left[\sum_{k=1}^{L^{\left(2\right)}}‍h_{k}^{\left(2\right)}W_{k}^{\left(3\right)}\right]$$ (4)

where *W*^(2)^ is a matrix of *L*^(2)^ × 1 weights and σ is the softPlus activation function (*98*)

$$\sigma \left(z\right)=\text{log}\left(e^{z}+1\right)$$ (5)

that ensures that the output of the network, which is the rate, is a positive number. Last, the baseline rate is transformed into the species-time–specific speciation or extinction rate, through an output layer

$$\lambda^{\left(i,t\right)}=\varphi \left(\lambda_{b}^{\left(i,t\right)},t_{\text{reg}}\right)$$ (6)

where φ is a regularizing function

$$\varphi \left(x,t_{\text{reg}}\right)=\frac{x^{t_{\text{reg}}}}{E\left(x^{t_{\text{reg}}}\right)}\;E\left(x\right)$$ (7)

with *t*_reg_ ∈ [0,1] as a regularizing parameter and E( · ) being the arithmetic mean function averaging the baseline rates across all taxa and time bins. This function does not alter the mean rate across time and taxa compared with the baseline rates [i.e., E(λ_b_) = *E*(λ)] but has the property of shrinking the rates around the mean for *t*_reg_ values closer to 0, while leaving them unaltered if *t*_reg_ ≈ 1. The rates become constant through time and across species when *t*_reg_ = 0. The neural network can be configured with different number of hidden layers and nodes and can include a bias node (or intercept) in the last hidden layer. Its parameters (the weights) are shared among all species in the dataset and all time bins, and two independent networks are used to obtain speciation and extinction rates with parameters $W_{\lambda }=\left\{W_{\lambda }^{\left(1\right)},W_{\lambda }^{\left(2\right)},W_{\lambda }^{\left(3\right)}\right\}$ and $W_{\mu }=\left\{W_{\mu }^{\left(1\right)},W_{\mu }^{\left(2\right)},W_{\mu }^{\left(3\right)}\right\}$ , respectively, while a single regularizing parameter *t*_reg_ is used for both.

Unlike in other birth-death models implemented in PyRate, the parameters sampled through MCMC under this model are not directly the speciation and extinction rates, but rather the weights of the two neural networks, **W**_λ_ and **W**_μ_ and the regularizing parameter *t*_reg_, from which the rates are derived for each species and each time bin (Eqs. 1 to 4). The number of parameters in the model depends on the number of predictors *J* (which at minimum include time but can incorporate categorical and continuous traits and time-dependent variables) and the network architecture (i.e., number of layers and nodes). The neural networks used in the BDNN model are unsupervised as they are not trained against ground truth speciation and extinction rates but used within PyRate’s Bayesian framework, in which speciation and extinction rates are used to calculate the likelihood of the observed fossil occurrence data (along with the parameters of the preservation process). The likelihood of an extinct lineage *i* with estimated origination time *s_i_* and extinction time *e_i_* and predictors *x_i_* is based on the sampled birth-death process (*52*)

$$P\left(s_{i},e_{i},x_{i}|W_{\lambda },W_{\mu }\right)\propto \lambda^{\left(i,s_{i}\right)}\times \mu^{\left(i,e_{i}\right)}\times \text{exp}\left[-\int_{s_{i}}^{e_{i}}‍\lambda^{\left(i,t\right)}+\mu^{\left(i,t\right)}dt\right]$$ (8)

which for extant lineages (i.e., with *e_i_* = 0) reduces to

$$P\left(s_{i},x_{i}|W_{\lambda },W_{\mu }\right)\propto \lambda^{\left(i,s_{i}\right)}\times \text{exp}\left[-\int_{s_{i}}^{0}‍\lambda^{\left(i,t\right)}+\mu^{\left(i,t\right)}dt\right]$$ (9)

The BDNN model uses the same algorithm implemented in other PyRate models (*52*) to sample the times of origination and extinction of all species, preservation rates, and the weights of the neural networks from their joint posterior distribution. We assigned standard normal distributions, 𝒩(0,1), as regularizing priors on the weights (*71*), and sampled these weights through MCMC with normally distributed proposal kernels. We used a truncated exponential prior on the regularizing parameter *t*_reg_ ∼ Exp_*T* = 1_(1), such that the highest prior probability was assigned to *t*_reg_ = 0, thus favoring a null hypothesis of constant rates, while the lowest nonzero prior probability was assigned to *t*_reg_ = 1, which corresponds to no regularization of the baseline rates.

After preliminary analyses using simulated datasets and the proboscidean data, we found negligible differences among neural network architectures and chose to use two hidden layers with 16 and 8 nodes. These settings worked well for our datasets with 200 to 300 species and 6 to 11 predictors. However, we encourage users to experiment with different network architectures to assess the results’ robustness to different model configurations.

### Postprocessing

We implemented a number of postprocessing steps using the output of a BDNN analysis to identify whether traits and time-variable predictors affect speciation and extinction rates, the direction of their effects, and their importance rank. This is important because normal model comparison to select the predictors (e.g., using Bayes factors) is impractical because of the high number of possible combinations.

We used PD plots (*53*) to visualize the magnitude and shape of the inferred effect of traits and time-variable predictors on rates of speciation and extinction. PD plots are widely used in interpretable machine learning (*54*) and show the marginal effect of one or two predictors on the outcome of a machine learning model by averaging across the remaining predictors.

As neural networks do not directly provide a measure of predictor importance, we implemented three approaches to rank the predictors, quantifying different aspects of importance: influence on model fit through feature permutation, credible rate differences in PD plots, and the change induced in speciation or extinction rate by a predictor through SHAPs. Preliminary analyses using simulated fossil records with known predictors of rate variation showed that they could not always be correctly identified using a single approach. Accordingly, we ranked the predictors’ importance on the basis of the three approaches and used the QUICK algorithm (*57*) to obtain the consensus ranking across them as an overall measure of importance. We separately ranked the individual effects and the interaction strength between two predictors because strong individual effects may imprint interactions and therefore overshadow the importance measures of other individual predictors.

For the first measure of predictor importance, we quantified the decrease in birth-death likelihood after permuting the predictor’s values. This differs slightly from the original implementation of feature permutation (*99*) in targeting model fit instead of prediction error (which is typically expressed as negative log-likelihood). An interaction between two predictors is considered important if the decrease in model fit after permuting both is greater than the sum of the individual reductions when only one of the predictors is permuted.

Second, we derived PD-based posterior differences in rates as a measure of predictor importance. We first identified the maximum and minimum of the mean rate (i.e., averaged over MCMC generations) along the predictor’s range of values. We then quantified how often the rates at the maximum point exceeded those at the minimum point across the MCMC samples.

Last, we used SHAPs (*55*) to quantify the contribution of each predictor toward the speciation and extinction rates. Specifically, we obtained SHAP values using the κ-additive Choquet integral–based method (*100*) because it is computationally more efficient than the original Kernel SHAP and quantifies the importance of the interaction between two predictors. The SHAP values also indicate the extent to which each predictor has leverage on the speciation and extinction rate of each species. This aids the interpretation of species-specific rates because, in a neural network, as used for the BDNN model, the direction and strength of a predictor’s influence on individual rates cannot be readily obtained from the inferred network’s weights.

### Simulation scenarios

We simulated fossil datasets and species traits under nine birth-death scenarios where speciation and extinction rates are influenced by traits, time, environmental change through time, or their combination. All diversification scenarios were simulated forward in time at discrete time steps of 0.01 Ma, starting at the same root age of 35 Ma. To avoid extremely species-poor or species-rich datasets, we targeted an output of 200 to 300 extant or extinct species over this period. In all diversification scenarios, species were characterized by one continuous trait and one discrete trait with two states, even in scenarios where speciation and extinction were not affected.

We simulated the evolution of the continuous trait, starting with a value of zero, by an unbiased random walk with a rate of σ^2^ = 0.02 (i.e., the equivalent to Brownian motion in continuous time). In this standard model of continuous trait evolution, a descendant species inherits the complete phenotype from its ancestor; thus, there is no trait change at cladogenetic speciation events. However, species in the fossil record are typically defined on the basis of their morphology [e.g., *101*)], which requires some phenotypic changes during speciation and relatively few anagenetic changes. This corresponds to the classic microevolutionary view of trait divergence at speciation (*25*, *102*). While the interconnection between the morphological species concept and phenotype makes it difficult to test this mode of trait evolution from the fossil record, trait shifts at speciation events have been demonstrated along the phylogeny of extant species (*103*). We added such an instantaneous phenotypic change by drawing a new trait value for the descending species from a normal distribution with a mean equal to the trait value of the ancestor and a standard deviation (SD) of $\sqrt{0.2}$ . The evolution of the categorical trait was simulated in a similar way as for the continuous trait. There was no anagenetic transition between the two states, but a probability of *P* = 0.1 for a state change at speciation.

The diversification scenarios differed in the dynamics of speciation and extinction rates through time and whether these depend on the species’ traits or paleotemperature (fig. S1). The first scenario featured constant speciation and extinction rates set to λ = 0.2 and μ = 0.1, respectively.

Scenario 2 included a sigmoidal change in rates where speciation decreased from 0.4 at the beginning of the diversification process to 0.1 at the present, while extinction increased over time from 0.05 to 0.4. The rates followed a logistic function with midpoint set at 20 and 15 Ma (for speciation and extinction, respectively) and steepness set to 0.5.

Scenario 3 imposed two instantaneous rate shifts. The speciation rate was set to decrease from 0.4 to 0.1 at 20 Ma and from 0.1 to 0.01 at 10 Ma. The extinction rate peaked at 0.3 from 15 to 10 Ma, while earlier the rate was 0.05 and thereafter 0.01.

Under scenario 4, global temperature through time exerts influence on speciation and extinction. To explore the ability of the neural network to capture a nonmonotonic relationship between a predictor and rate and an interaction between two rate predictors, we simulated a bell-shaped link between temperature and rate for the first state of the categorical trait and an inverted bell curve for the second state (i.e., Gaussian functions; see fig. S1D). We derived the temperature trajectory for the time frame of the simulation from isotope data (*104*) using the equations provided by Hansen *et al.* (*105*) and scaled it to an SD of 1. The peak of the bell was set to the mean temperature over the time frame of the simulation (i.e., 17.7°C) and corresponded to a baseline speciation rate of 0.5 and an extinction rate of 0.4. We parameterized the rate change with warmer or cooler temperatures through a given SD for the Gaussian function between the temperature and rate. Effectively, this transforms the bell to be more peaked or flatter and less divergent from the baseline rate with a high or lower SD, respectively. We set the SD of the Gaussian function to 1.2. Thus, an increase or decrease of 2.1°C from the mean of the temperature trajectory (i.e., 1 SD) resulted in a 50% lower rate than the baseline, which then decreases to 3% when the temperature difference is 2 SDs, and asymptotically reaches a rate of zero with even higher temperature deviations. For the second state, the relationship between temperature and rate is inverted, with a rate of zero at the mean temperature and an asymptotically increase toward the baseline rate by higher and lower temperatures.

In the trait-dependent diversification scenario 5, species featuring the first state of the categorical trait were characterized by a speciation rate of 0.1 and an extinction rate that increased over the simulated 35 Ma from 0.01 to 0.1, resulting in equal rates at the present. Species of the second state had 5-fold higher rates, which means that the strength of the increase in extinction was state dependent and therefore constitutes an interaction between the categorical trait and time.

Under scenario 6, the species’ continuous trait affected its speciation and extinction rate through a Gaussian function, resulting in that both rates are highest at an (arbitrary) trait value of 0 and decreasing with increasing positive or negative values. We set the Gaussian function such that a change of 1 or 2 SDs from the initial trait value of 0 reduced the baseline rate (here set to 0.5 for speciation and 0.4 for extinction), by 70 and 20%, respectively.

In scenario 7, speciation and extinction rates were determined by the interaction between continuous and categorical traits. While the rates for species having the first state of the categorical trait were obtained from the same bell-shaped function as in scenario 6, species of the second state had an inverted relationship between continuous trait and rate: Trait values of 0 defined a rate of 0, and baseline rates are asymptotically approached with increasingly smaller or larger trait values.

Scenario 8 featured an interaction between time and the continuous trait. We set at 15 Ma the time of the switch between a bell-shaped function and an inverted bell function, correlating the continuous trait with speciation and extinction rates.

Last, scenario 9 was based on the datasets simulated under scenario 6, i.e., with rates dependent on a continuous trait, which was, however, omitted from the subsequent BDNN analysis. An additional trait was instead included on the basis of a random draw from a standard normal distribution, maintaining the same number of predictors.

We simulated 100 datasets of fossil occurrence under each scenario, assuming a time-variable Poisson process of fossilization and sampling, with independent rates drawn for each geological stage randomly from *q_i_* ∼ 𝒰[log(0.5), log(5)]. We additionally implemented preservation rate variation among species by drawing species-specific multipliers from a gamma distribution with shape and rate parameters set equal and drawn from α ∼ 𝒰[log(0.5), log(5)]. The preservation rate for each species is then obtained as the product between the baseline rates *q* and the species-specific multipliers. This model reflects the time-variable Poisson process with rate variation among lineages implemented in PyRate (*52*), with a parameterization similar to what we estimated from the proboscidean dataset. While the stochastic evolution of the continuous trait was tracked for every species throughout the simulation, the value used as input for the BDNN inference was set as the average of the trait only at the sampled occurrences times of the species, thus reflecting the type of morphological information observable in empirical datasets. We approximated the phylogenetic relatedness of species by decomposing the simulated trees into eigenvectors based on the phylogenetic pairwise distance matrix among the tips of the tree. This approach, as implemented in the PVR 0.3 (*106*) package for the R 4.3 statistical programming environment (*107*), results into a numerical representation of the tree capturing both topology and branching times (*56*).

The input data used in our analyses included (i) the fossil occurrences, (ii) the simulated traits, and (iii) time itself and the first two phylogenetic eigenvectors. In scenario 4, we additionally included paleotemperature as a time-dependent variable. Conversely, the times of rate shift implemented in scenarios 2, 3, and 8 and the predictive trait in scenario 9 were not included among the predictors. Time and phylogenetic eigenvectors were therefore expected to capture rate variation driven by unobserved traits or rate shifts.

### Analysis of simulated data

We analyzed the ability of the BDNN model to capture the simulated effects on speciation and extinction by comparing the results with those obtained under the birth-death-shift (BDS) model, which infers instantaneous rate shifts over time and is widely used in exploratory analyses of diversification dynamics from fossil data [e.g., (*52*)]. For both models, we ran 1,000,000 MCMC iterations and sampled every 1000th iteration after a burn-in of 25% to obtain the posterior parameter distribution. We also analyzed the data under the boundary-crosser method (*26*, *108*), providing a comparison of the performance of the BDNN model against an approach based on different underlying models and implementations. We obtained boundary-crosser speciation and extinction rates using the R package divDyn 0.8.2 (*109*) using time bins of 2 million years. We additionally performed the analyses on 100 bootstrapped datasets to estimate confidence intervals around the rate estimates (*26*), which we used to approximate the coverage as the fraction of simulations in which the true rates were included in the confidence interval.

We quantified the overall accuracy of the inferred species-specific speciation and extinction rates as the median absolute relative error, which was calculated as

$$\begin{array}{cc} X & =\left(\frac{r_{1}-{\hat{r}}_{1}}{r_{1}},\frac{r_{2}-{\hat{r}}_{2}}{r_{2}},\ldots ,\frac{r_{n}-{\hat{r}}_{n}}{r_{n}}\right)\\ \text{Median absolute relative error} & =\frac{X_{\frac{n}{2}}+X_{\frac{n}{2}+1}}{2} \end{array}$$ (10)

where *r_i_* is the true rate at origination or extinction of a simulated species, ${\hat{r}}_{i}$ the inferred species-specific rate at the inferred time of origination or extinction, *X* contains the ordered absolute percentage errors, and *n* is the even number of replicates. Thus, for instance, an error of 0.2 represents a median deviation of 20% of the true value. We opted for the median instead of the mean absolute error because the latter can be overinflated by relative errors when the true rates are close to zero (fig. S1, F to H). We defined the true speciation rate of a species as the rate assigned to its parent species, which may differ from that of the descendant because of, for instance, trait differences between them. Because the BDS model does not infer species-specific rates but rates through time, we intersected them with the inferred times of origination and extinction of each species to obtain and compare them with the species-specific rates of the BDNN model.

### Proboscidean fossil record, traits, and paleoenvironment

We used a recently compiled fossil occurrence dataset of proboscideans (*58*) from which we excluded 10 species. Five species belonged to a monophyletic group and were represented only by singleton occurrences more than 10 Myr older than the other species, resulting in highly uncertain rates before 40 Ma in an exploratory diversification analysis (*58*). The remaining five species had no trait information. The final dataset included 2118 occurrences for 175 proboscidean species. To account for age uncertainties in fossil occurrences, we resampled the occurrence ages randomly from uniform distributions spanning their temporal ranges and generated 10 replicated datasets (*52*).

Cantalapiedra *et al.* (*58*) summarized 17 ecomorphological traits by an ordination into two NMDS axes, which we used in our BDNN analysis. Dental masticatory durability is captured by the first axis, which broadly correlates with the change from a browsing-specialized diet to a more generalized diet capable of processing a higher proportion of grass and even wood. The second trait axis summarized craniodental modifications such as from a flat to a short and high skull. While the adequacy of variables summarizing multiple traits through dimensionality reduction techniques (e.g., NMDS and principal components analysis) in evolutionary analyses remains debatable, potential issues relate to our ability to model how these summarized traits themselves evolve (*110*, *111*). In contrast, here, we used them as predictors of speciation and extinction rate variation without attempting to infer the modes of their evolution.

Because of shared evolutionary history among species, traits and geographic distribution exhibit phylogenetic inertia (*112*). Not accounting for the resulting statistical nonindependence could inflate the signal of trait- or geography-dependent speciation and extinction rates. We thus incorporated the first two phylogenetic eigenvectors, calculated from a tree of living and extinct proboscideans (*58*) as a proxy for phylogenetic relatedness, and included them as species-specific features in addition to traits and geographic distribution in the BDNN inference. To account for phylogenetic uncertainties, we recalculated the eigenvectors for a different tree (randomly sampled from their posterior distribution) for each of the 10 replicates with resampled fossil ages.

We also compiled geographic distribution for all proboscidean species based on (*58*), with the distribution categorized into Africa, Americas, and Eurasia or whether the species is an island endemic. On the basis of the geographic distribution of each species, we obtained an individual time series of paleoenvironmental conditions. As our first paleoenvironmental measure, we recorded the origin of open and grass-dominated habitats as a binary predictor based on (sub)continent-specific timelines inferred from paleobotanical records (*90*). We defined the emergence of these habitats in Africa at 15.97 Ma and in Eurasia at 20.44 Ma, except for Indian species for which we used an onset at 11.63 Ma. While in South America open and grass-dominated habitats date back to the Eocene, we used the North American age of 23.03 Ma for the Americas because all South American proboscideans are much younger. Second, spatially explicit reconstructions of climate through time were used to obtain species-specific time series of paleotemperature based on the species distribution ranges. Because climate circulation models (CCM) cover only a fraction of the proboscidean diversification history, we used a vegetation-derived climate reconstruction (*94*) with a temporal resolution of ∼0.17 Ma and a spatial resolution of 2° × 2°. For the Last Glacial Maximum and the Last Interglacial, temperatures were extracted from CCMs (*113*, *114*) because a higher temporal resolution is needed to compare the potential effects of temperature and humans on extinction rates. Because there is only a limited number of fossil occurrences per time bin, we used all fossil occurrences of species regardless of their age to approximate species distribution ranges. We constructed a convex hull linking all countries of a continent where fossils of the species were found and did not attempt to trace changes in distribution through time. The paleo-position of the convex hulls was reconstructed for each time bin of the paleo maps using the rgplates 0.2.1 (*115*) interface to the GPlates desktop application (*116*) and used to extract the mean paleotemperature across the approximated distribution with the R package terra 1.7.29 (*117*). All spatial data were transformed into the Mollweide equal-area map projection to avoid bias in temperatures due to high-latitude distortion of geographic coordinates. We used the following boundaries to bin paleotemperatures: Oligocene (33.9 Ma), Burdigalian (20.44 Ma), Langhian (15.97 Ma), Serravallian (11.63 Ma), Tortonian (7.246 Ma), Messinian (5.333 Ma), Pliocene (2.58 Ma), Calabrian (1.8 Ma), the Mid-Pleistocene transition (∼0.8 Ma), Late Pleistocene (0.129 Ma), and the Holocene (0.0117 Ma). These boundaries define when speciation and extinction rates are allowed to change over time. While we used bins with a width of 1 Myr in our simulations, we opted here for an increasing temporal resolution toward the present because it reflects the higher confidence in paleotemperature reconstruction, the lower uncertainty in fossil ages, and the resolution needed to inform the model of human appearance. In a previous study on proboscidean diversification over time, there was no evidence of rate shifts before the Messinian (*58*), and thus, the relatively coarse resolution used here is unlikely to conceal temporal changes in diversification dynamics. We encoded the time of origin or arrival of early and modern humans on each continent and associated islands in the respective time bins using two levels. Specifically, we considered the proboscidean overlap with modern humans as ubiquitous in the most recent time bin (i.e., the Holocene). For African and Eurasian species, we also included the overlap with modern humans in the penultimate bin (i.e., the Late Pleistocene) and with early humans in all other time bins younger than 1.8 Ma.

### BDNN analysis of the proboscidean data

We z-transformed the continuous traits, phylogenetic eigenvectors, and paleotemperature to a mean of zero and an SD of 1. For all 10 replicates with resampled fossil ages, we ran 50,000,000 MCMC iterations and sampled every 10,000th iteration after a burn-in of 25% in PyRate. We used a preservation model that allows for heterogeneity in fossil sampling among species and through time (*52*). The analyses were completed in approximately 48 hours on standard AMD central processing units. After the inference, we applied all postprocessing steps described above using 1000 samples from the posterior distribution for each replicate. In addition, we conducted a combined analysis by concatenating 100 samples from each of these replicates.

To determine whether the rate heterogeneity inferred by the BDNN model exceeded the expectation under a constant-rate birth-death process, we simulated 100 datasets matching the empirical data in terms of number of traits, clade age, and approximate number of species (range 175 ± 52). We the determined the 95% quantile of coefficient of variation among species-time–specific rates and compared it with the coefficient of variation obtained from the proboscidean dataset.

We also reanalyzed the dataset under a simpler set of variables, including only time, phylogenetic eigenvectors, paleoclimate, insularity, body mass (discretized into eight bins), and human overlap. This allowed us to assess the consistency between the two analyses and whether body mass alone could capture most of the signal encapsulated by the two NMDS axes.

*Note added in proof*: After acceptance, the authors became aware of a recently published paper (*118*).This added reference adds an empirical example to theoretical foundations.
