# Supplementary material for: Trait-mediated speciation and human-driven extinctions in proboscideans revealed by unsupervised Bayesian neural networks
Source: Sci Adv. 2024 Jul 24;10(30):eadl2643. doi: 10.1126/sciadv.adl2643 (PMC11268411; doi:10.1126/sciadv.adl2643)
Supplement: Supplementary file 1 — Figs. S1 to S8 Tables S1 to S3 Legends for data S1 and S2 [file sciadv.adl2643_sm.pdf]

Science Advances
AAAS

Supplementary Materials for

Trait-mediated speciation and human-driven extinctions in proboscideans revealed by unsupervised Bayesian neural networks

Torsten Hauffe *et al.*

Corresponding author: Torsten Hauffe, torsten.hauffe@gmail.com; Daniele Silvestro, daniele.silvestro@unifr.ch

Sci. Adv. **10**, eadl2643 (2024)
DOI: 10.1126/sciadv.adl2643

The PDF file includes:

Figs. S1 to S8
Tables S1 to S3
Legends for data S1 and S2

Other Supplementary Material for this manuscript includes the following:

Data S1 and S2

Supplementary figures

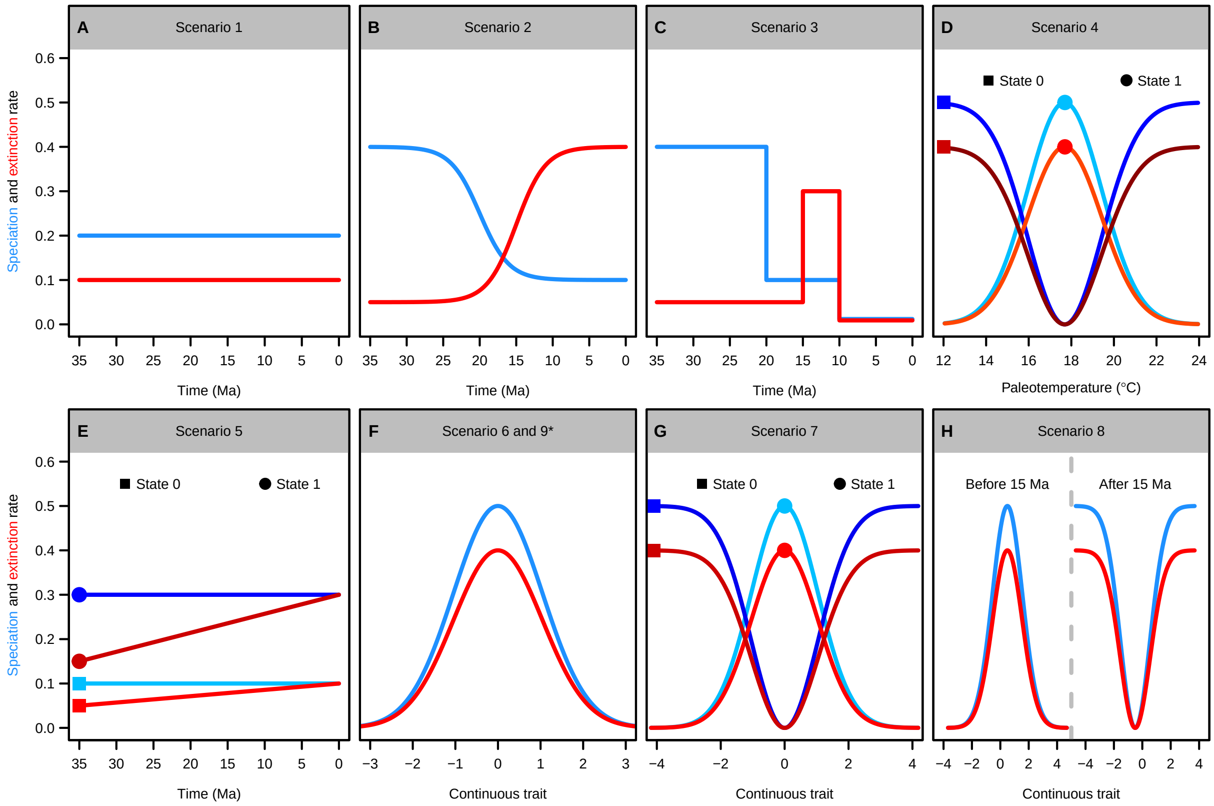

Fig. S1. Simulated diversification scenarios. To assess the performance of the BDNN model, we specified eight scenarios where speciation and extinctions rate: (**A**) are constant through time and independent of species' traits, (**B**) change over time according to a logistic function, (**C**) shift instantaneously at discrete moments in time, (**D**) depend on two states of a categorical trait that determine whether rates are a bell-shaped or inverted bell-like function of paleotemperature, (**E**) approach a state-dependent equilibrium due to an increase in extinction over time, (**F**) are defined by a non-monotonic function of a continuous trait, (**G**) will show a bell-shaped or inverted bell-like relationship with a continuous trait, depending on the state of a categorical trait, or (**H**) change from a inverted bell-shaped function of a continuous trait to a bell-shaped relationship at 15 Ma. All simulated datasets include additional traits and time-dependent variables that did not influence speciation and extinction rates. For scenario 9, where the influential trait is missing, we used the data of scenario 6 and replaced the continuous trait with a random draw from a standard normal distribution. All rates are given in units of events $\cdot$ lineage^{-1} $\cdot$ myr^{-1}.

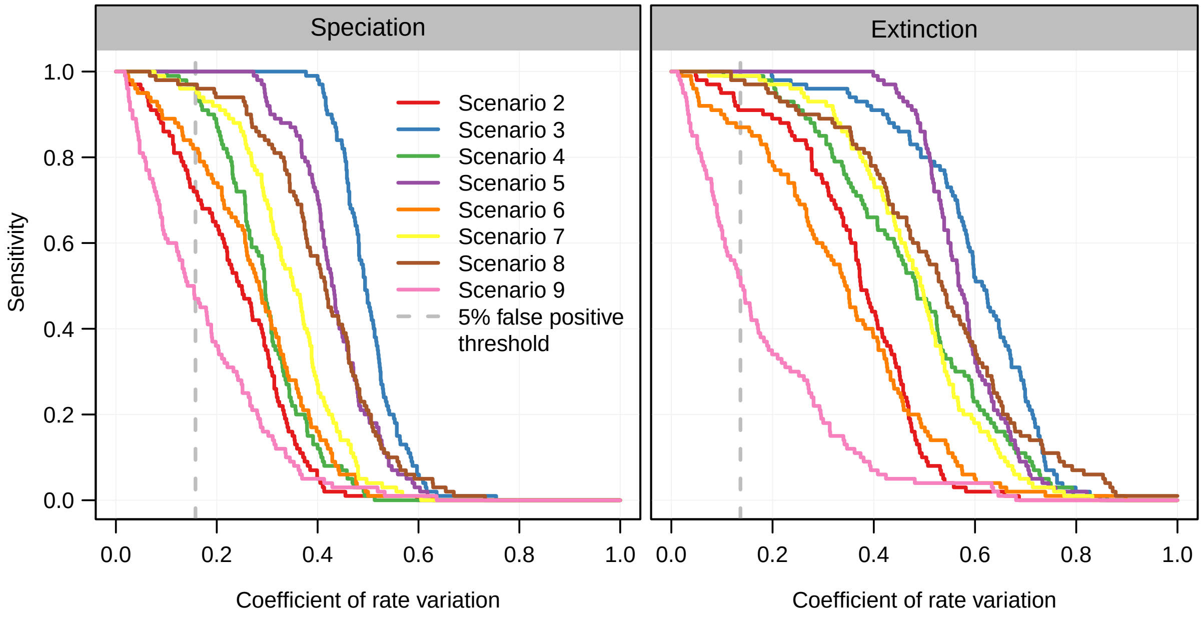

Fig. S2. Sensitivity to find evidence of rate variation among species and over time. For simulated diversification scenarios where speciation and extinction rates varied as a function of traits and time-dependent variables, we quantified the coefficient of variation in inferred lineage-specific rates and calculated over 100 thresholds the proportion of correctly evidenced rate heterogeneity. The dashed vertical line displays the threshold of 0.16 for speciation and 0.14 for extinction to detect rate variation.

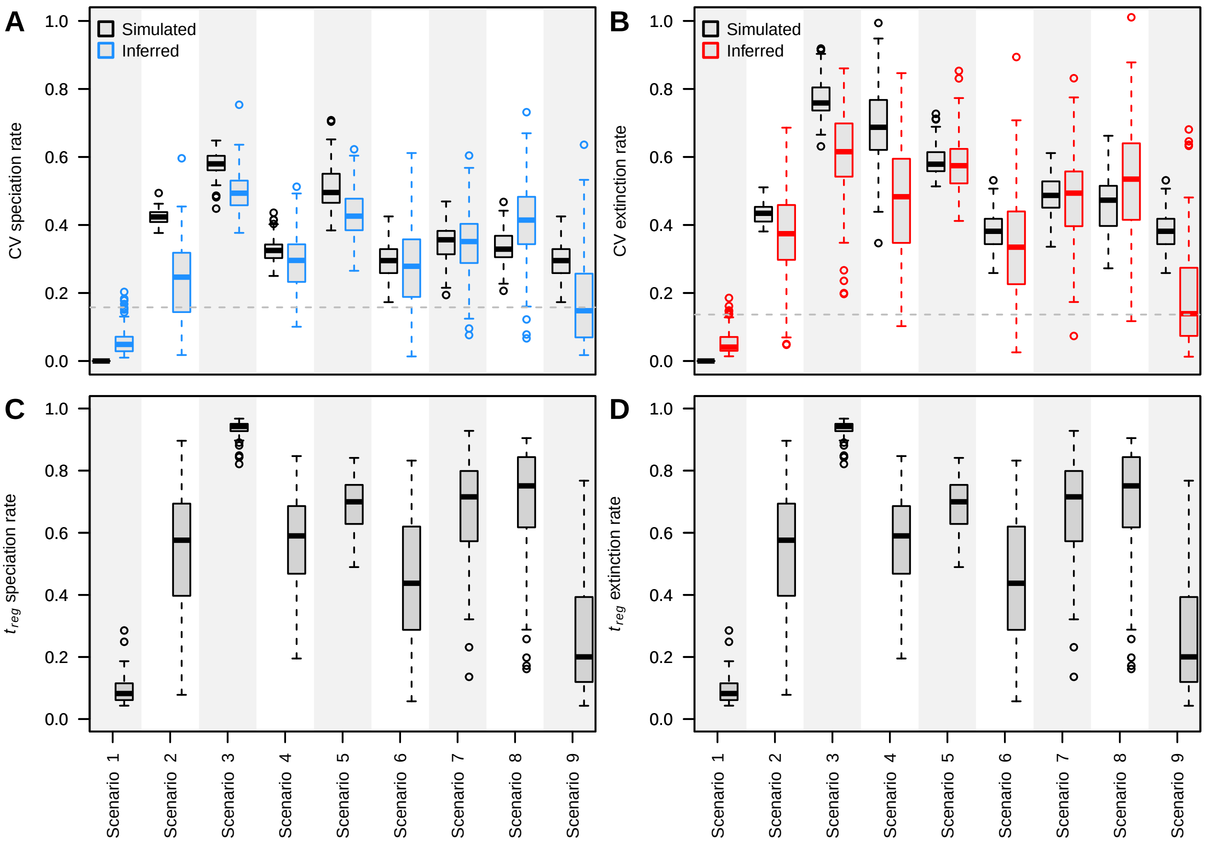

Fig. S3. Simulated and inferred variation in lineage-time-specific rates. Coefficient of variation (CV) in (**A**) speciation and (**B**) extinction rate across all species was calculated in each of the 100 simulation replicates under eight scenarios of diversification depending on traits and time-dependent variables. The dashed horizontal lines display the thresholds for speciation and extinction above which a constant rate model was rejected with 95% specificity. The simulated speciation rate was taken from the ancestral lineage from which the species branch off and whose rates are inferred. Regularization parameter t_{reg} for (**C**) speciation and (**D**) extinction rate shows the degree of shrinking lineage-time-specific rates to a common mean in the neural network. Rates are constant when $t_{\text{reg}} = 0$ and not shrank when $t_{\text{reg}} \approx 1$.

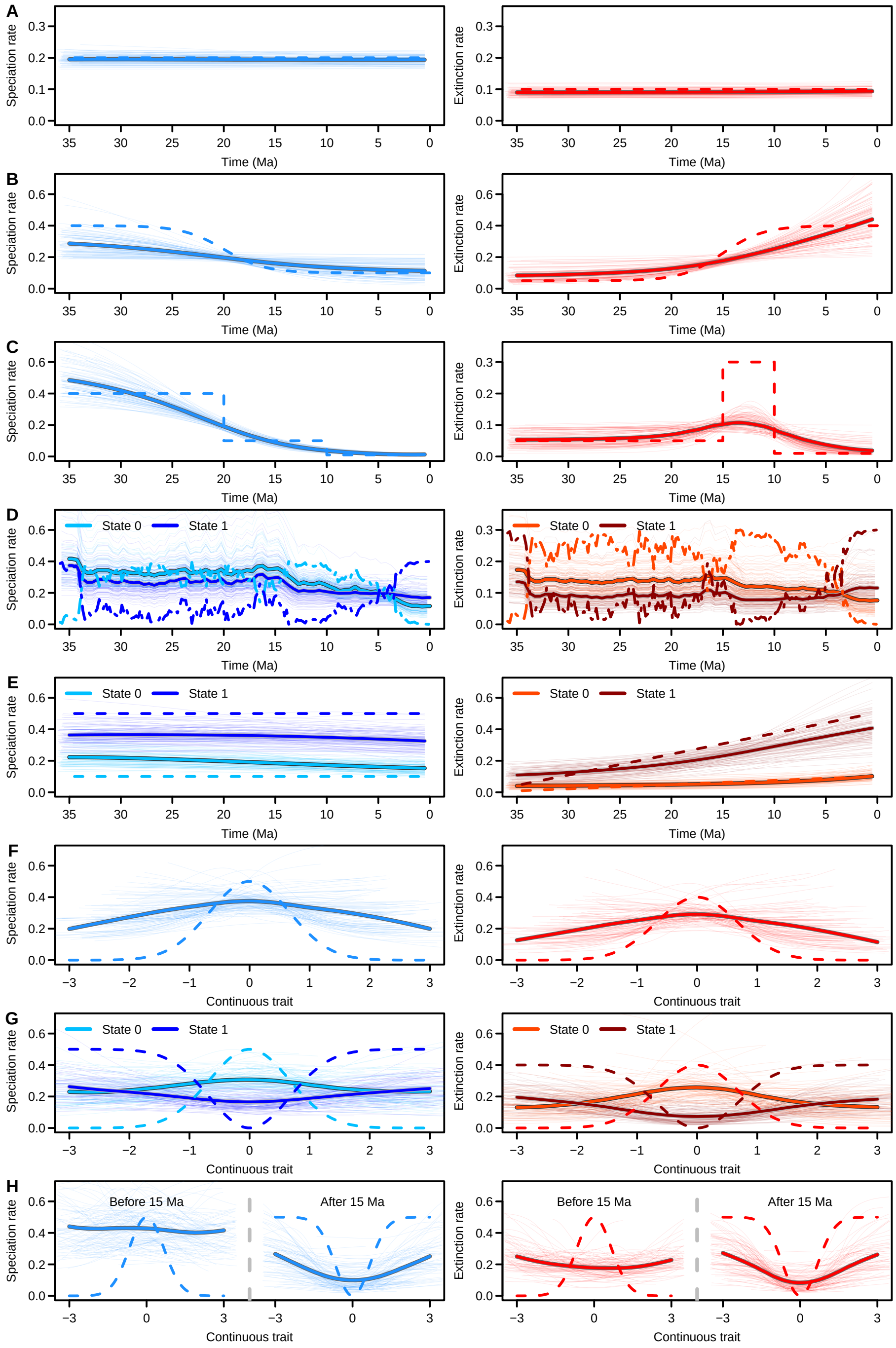

Fig. S4. Simulated versus inferred rates. Under eight scenarios **A–H** (see Fig. S1), true speciation and extinction rates (dashed lines) were a function of traits and time-dependent variables and used to simulate 100 fossil datasets for each scenario. Transparent lines show partial-dependence (PD) rates calculated from the inferred BDNN model and the thick solid line represents the average across them after locally estimated scatterplot smoothing (loess). Each simulated dataset subjected to the BDNN inference contained complementary traits, phylogenetic eigenvectors, or time-dependent variables that did not influenced diversification and the PD rates display the exclusive influence of the focal traits and variables by marginalizing over the complementary ones. All rates are given in units of events $\cdot$ lineage^{-1} $\cdot$ myr^{-1}.

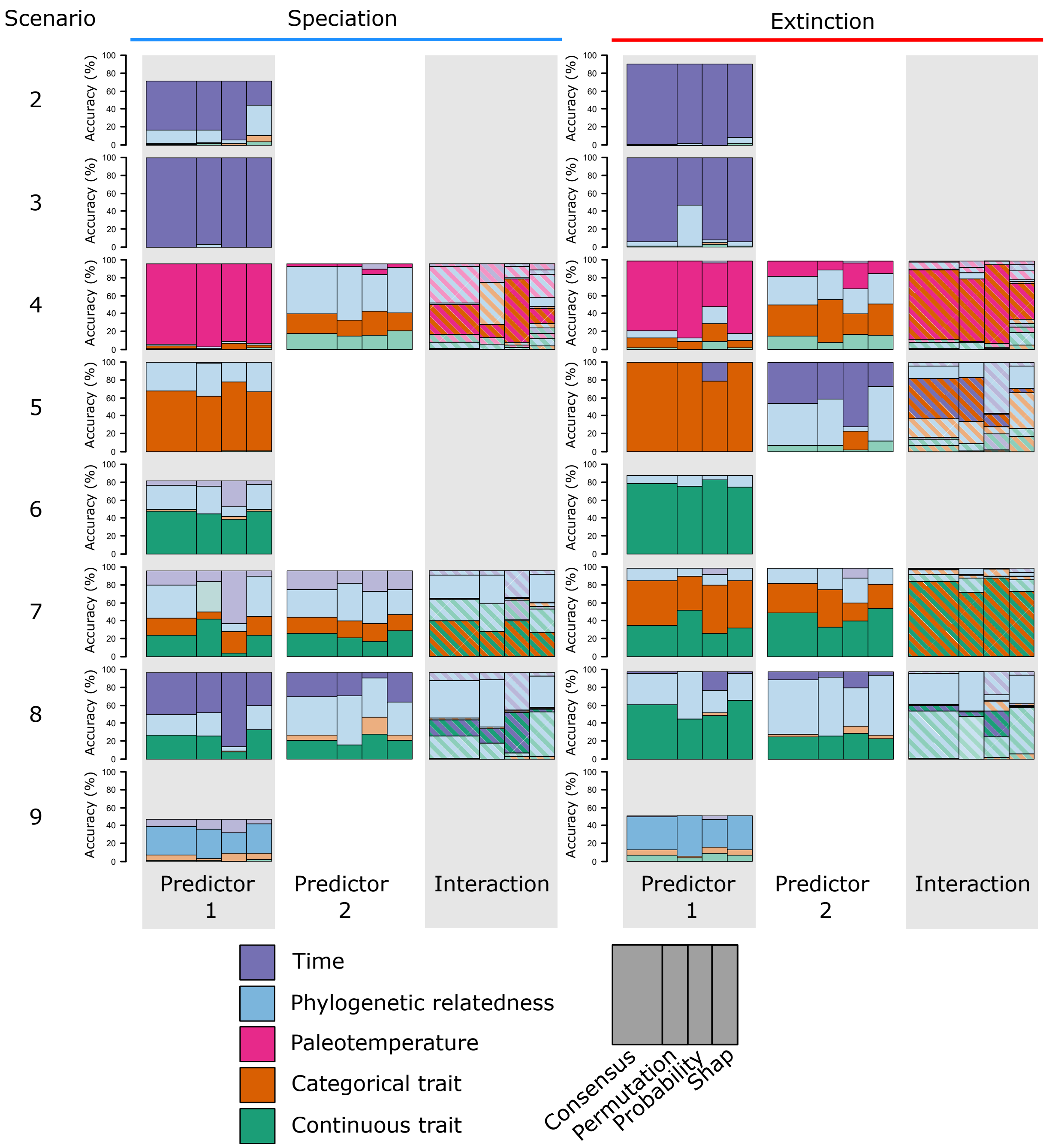

Fig. S5. Power to identify rate predictors. Barplots show the result of three individual metrics from explainable artificial intelligence and their consensus. Solid colors display the proportion when a simulated effect of traits and time-dependent variables on speciation and extinction rates was actually ranked as the most influential (*Predictor 1*) during the BDNN inference, among the top two (*Predictor 2*) when one rate determinant alters the effect of another, or in case of scenario 9 phylogenetic relatedness identified instead of an omitted influential trait. The share of a two-way interaction being identified as the most important is indicated by the hatched pattern (*Interaction*). Total height of the barplots equals the number of simulations that exceed our threshold to reject the constant rate model.

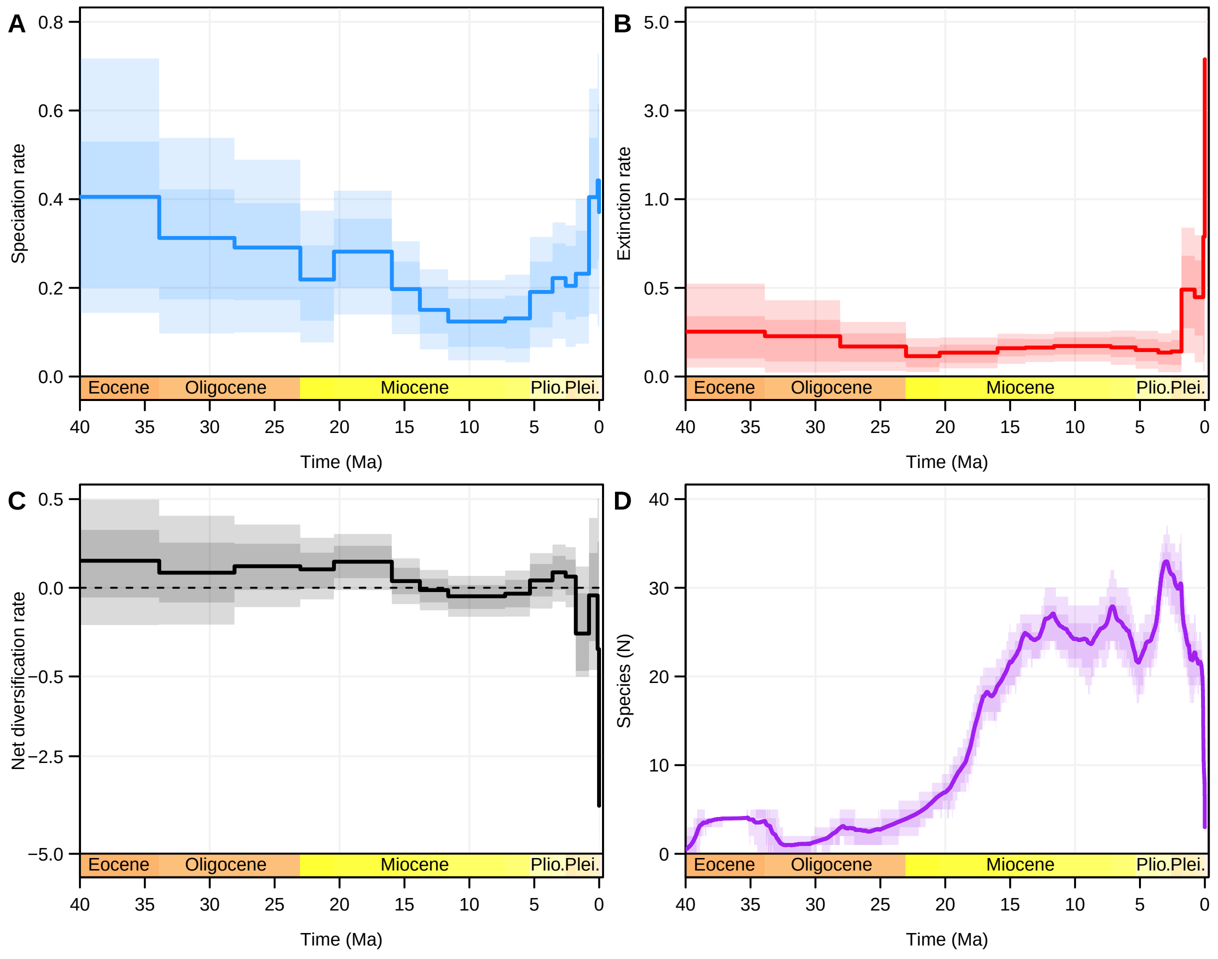

Fig. S6. Proboscidean diversification through time. Shown are (**A**) speciation rate, (**B**) extinction rate, (**C**) net-diversification, and (**D**) diversity across 10 replicates, incorporating the dating uncertainty of the fossil record. Solid lines display the mean and the shaded interval the 75% and 95% credible interval. The y-axis of the extinction and net-diversification rate is compressed for values greater than 1.0 and smaller than 0.5, respectively, to aid rate comparison before the Holocene extinction peak. All rates are marginal rates, which were inferred with the BDNN model for all species that are extant in pre-defined time windows and are given in units of of events $\cdot$ lineage^{-1} $\cdot$ myr^{-1}. The diversity trajectory shows the range-through richness from the times of origin and extinction of all species that were inferred together with the speciation and extinction rates.

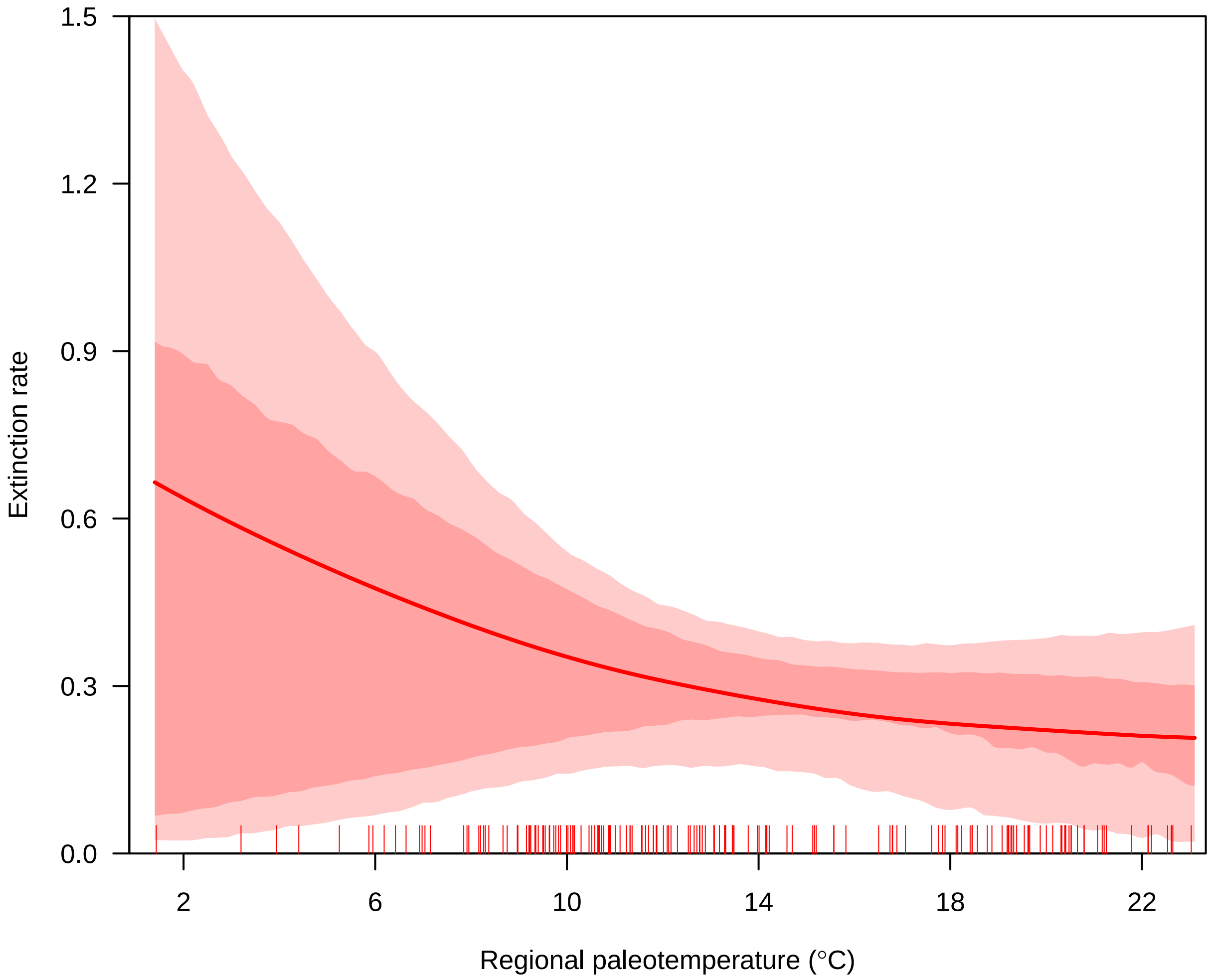

Fig. S7. Influence of regional paleotemperature on proboscidean extinction. Partial dependence plot showing the effect of paleotemperature on extinction rate. Solid lines display the mean partial dependence rate and the shaded interval the 75% and 95% credible interval. Ticks along the x-axis indicate the temperature at the inferred time of proboscidean extinction or the present-day value for the three extant elephant species. Extinction rate is given in units of of events $\cdot$ lineage^{-1} $\cdot$ myr^{-1}.

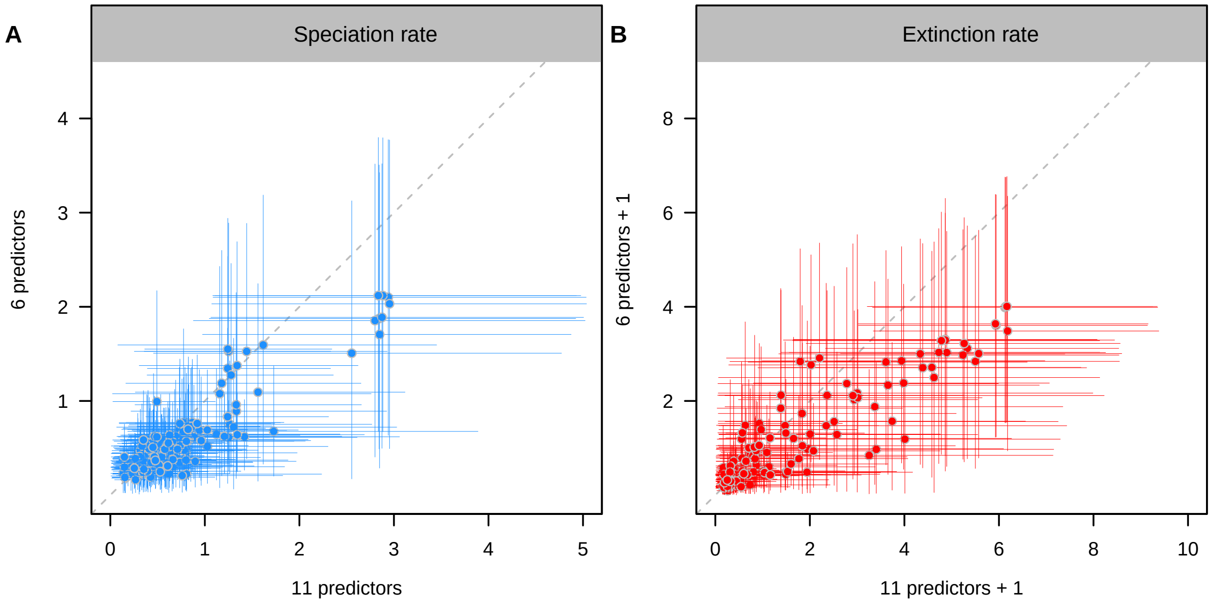

Fig. S8. Comparing proboscidean diversification models with different complexity. Species-times specific rates inferred for 175 proboscidean species based on 11 predictors (plus humans for extinction) plotted against a simpler BDNN model based on 6 predictors (plus humans for extinction). Circles represent the posterior mean rate, while the whiskers represent the 95% CI. The rates were considered consistent when the mean rate under the simpler model fell within the 95% CI of the more complex model. Speciation and extinction rates were consistent across 98% of the species, indicating that the models converge onto similar results overall.

Supplementary tables

Table S1. Comparing inferred rates. Across 100 replicated simulations under nine scenarios, we report the median absolute relative error for the comparison between the simulated and inferred rates, and the coverage indicating how often the 95% credible interval of inferred lineage-time-specific rates includes the true value. We used as true value the speciation rate of the ancestral lineage from which the lineage descended whose rate is inferred. All values show the mean and the respective standard deviation across simulations. Bold font highlights where the BDNN model outperformed the BDS inference according to the mean of the metric.

	Median absolute relative error						Coverage					
	Speciation			Extinction			Speciation			Extinction		
Scenario	BDNN	BDS	BC	BDNN	BDS	BC	BDNN	BDS	BC	BDNN	BDS	BC
1	**0.07**	0.07	0.24	**0.11**	0.11	0.35	**0.99**	0.96	0.83	**0.96**	0.87	0.70
	**±0.04**	±0.05	±0.10	**±0.06**	±0.07	±0.21	**±0.06**	±0.18	±0.13	**±0.12**	±0.33	±0.30
2	0.32	**0.15**	0.23	0.28	**0.17**	0.23	0.75	0.87	**0.91**	0.80	0.77	**0.88**
	±0.07	**±0.04**	±0.07	±0.06	**±0.06**	±0.06	±0.18	±0.12	**±0.08**	±0.15	±0.18	**±0.08**
3	0.38	**0.12**	0.30	0.66	**0.17**	0.33	0.74	**0.91**	0.69	0.44	**0.89**	0.61
	±0.04	**±0.07**	±0.11	±0.09	**±0.09**	±0.16	±0.06	**±0.14**	±0.11	±0.18	**±0.15**	±0.16
4	**0.29**	0.37	0.39	**0.48**	0.55	0.76	**0.81**	0.27	0.58	**0.57**	0.10	0.39
	**±0.05**	±0.07	±0.07	**±0.11**	±0.08	±0.13	**±0.11**	±0.14	±0.10	**±0.13**	±0.08	±0.15
5	**0.25**	0.54	0.58	**0.25**	0.57	0.58	**0.79**	0.02	0.35	**0.96**	0.06	0.46
	**±0.08**	±0.07	±0.08	**±0.07**	±0.05	±0.08	**±0.12**	±0.05	±0.11	**±0.05**	±0.09	±0.12
6	**0.23**	0.31	0.37	**0.25**	0.36	0.46	**0.80**	0.35	0.72	**0.80**	0.25	0.65
	**±0.05**	±0.07	±0.07	**±0.06**	±0.07	±0.08	**±0.14**	±0.14	±0.10	**±0.15**	±0.11	±0.11
7	**0.26**	0.40	0.43	**0.30**	0.49	0.55	**0.79**	0.30	0.59	**0.81**	0.16	0.51
	**±0.05**	±0.07	±0.08	**±0.06**	±0.07	±0.08	**±0.09**	±0.11	±0.12	**±0.11**	±0.08	±0.11
8	**0.27**	0.34	0.42	**0.32**	0.45	0.55	**0.80**	0.40	0.64	**0.78**	0.26	0.54
	**±0.06**	±0.09	±0.08	**±0.08**	±0.09	±0.10	**±0.10**	±0.14	±0.11	**±0.13**	±0.13	±0.11
9	**0.27**	0.31	0.37	**0.32**	0.36	0.46	0.65	0.35	**0.72**	0.57	0.25	**0.65**
	**±0.06**	±0.07	±0.07	**±0.07**	±0.07	±0.08	±0.22	±0.14	**±0.10**	±0.20	±0.11	**±0.11**

Table S2. Ranked predictors of proboscidean diversification. Post-processing output for the birth-death neural-network (BDNN) inference of proboscidean diversification dynamics over the past 40 Ma. It contains the ranked importance across three explainable artificial intelligence metrics expressing how speciation and extinction rates are influenced by proboscidean' eco-morphological trait axes (NMDS1 and NMDS2), phylogenetic relatedness (PVR1 and PVR2), geographic distribution (America, Africa, Eurasia, and Island), regional paleotemperature, time itself, and the spatial-temporal overlap with open-habitat grasslands and humans. The three metrics were the marginal probabilty, SHAP values, and the change in birth-death likelihood when permuting the predictor.

	Speciation				Extinction			
Predictor	Rank	Δ log likelihood	Marginal probability	SHAP value	Rank	Δ log likelihood	Marginal probability	SHAP value
NMDS1	1	−155.9	0.994	0.247	5	−88.5	0.779	0.168
NMDS2	7	−43.8	0.763	0.091	3	−95.3	0.943	0.173
PVR1	5	−49.7	0.721	0.107	6	−59.9	0.760	0.169
PVR2	4	−52.9	0.787	0.107	7	−76.6	0.734	0.153
Geography	3	−102.3	0.985	0.205	2	−141.1	0.957	0.274
Paleotemperature	6	−24.1	0.795	0.100	4	−26.2	0.883	0.210
Open grassland	8	−42.8	0.576	0.041	9	−66.0	0.633	0.054
Time	2	−139.6	1.000	0.148	8	−43.9	0.943	0.135
Humans					1	−91.3	1.000	0.762

Table S3. Ranked predictors of proboscidean diversification under a simpler set of eco-morphological traits. Post-processing output for the birth-death neural-network (BDNN) inference of proboscidean diversification dynamics over the past 40 Ma where speciation and extinction rates vary by body mass, phylogenetic relatedness (PVR1 and PVR2), insularity, regional paleotemperature, time itself, and the spatial-temporal overlap with humans. Structure and content equals Table S2.

	Speciation				Extinction			
Predictor	Rank	Δ log likelihood	Marginal probability	SHAP value	Rank	Δ log likelihood	Marginal probability	SHAP value
Body size	4	−27.7	0.678	0.091	4	−41.4	0.887	0.159
PVR1	5	−26.1	0.653	0.082	3	−55.5	0.838	0.175
PVR2	1	−55.9	0.994	0.159	2	−62.6	0.914	0.172
Island	2	−42.8	0.976	0.103	6	−24.8	0.820	0.069
Paleotemperature	6	−13.5	0.871	0.078	7	−13.8	0.683	0.107
Time	3	−30.2	0.849	0.058	5	−33.8	0.966	0.135
Humans					1	−49.2	0.999	0.451

Supplementary data

Data S1. Ranked predictors of proboscidean diversification. Post-processing output for the birth-death neural-network (BDNN) inference of proboscidean diversification dynamics over the past 40 Ma. It contains the ranked importance across three explainable artificial intelligence metrics and their summary statistics expressing how speciation and extinction rates are influenced by proboscidean' ecomorphological trait axes (NMDS1 and NMDS2), phylogenetic relatedness (PVR1 and PVR2), geographic distribution (America, Africa, Eurasia, and Island), regional paleotemperature, the spatial-temporal overlap with open-habitat grasslands and humans, and by time itself. The three metrics were the marginal probabilty, SHAP values, and the change in birth-death likelihood when permuting the predictor. Lower and upper values for the metrics indicate the 95% credible interval across 1000 Markov chain Monte Carlo samples. Main effects, indicated by no predictor in the second column, and interactions were ranked independently. As for categorical predictors, the third and fourth columns show which levels were compared. A BDNN analysis was conducted on ten replicates to account for the age uncertainty in proboscidean fossil occurrences and the resulting output was combined. The table contains 22 individual sheets, giving an overview of how the predictors affects speciation and extinction rates.

See data file: D ataS1_PredictorInfluenceProboscideans.xlsx

Data S2. Ranked predictors of proboscidean diversification under a simpler set of ecomorphological traits. Post-processing output for the birth-death neural-network (BDNN) inference of proboscidean diversification dynamics over the past 40 Ma where speciation and extinction rates vary by body mass, phylogenetic relatedness (PVR1 and PVR2), insularity, regional paleotemperature, the spatial-temporal overlap with humans, and by time itself. Structure and content equals Data S1.

See data ile: DataS2_PredictorIn luenceProboscideansSubset.xlsx
